# Reducing the availability of endogenous copper and glucose for cascade starvation therapy and chemodynamic therapy

**DOI:** 10.1016/j.mtbio.2025.101702

**Published:** 2025-03-24

**Authors:** Chunhui Wang, Pingting Ye, Mengyao Chen, Ruihao Li, Yixuan Wen, Yu Wang, Xiaohan Tong, Chunyan Dong, Shuo Shi

**Affiliations:** School of Chemical Science and Engineering, Breast Cancer Center, Shanghai East Hospital, Tongji University, Shanghai, 200092, PR China

**Keywords:** Copper chelator, Glucose oxidase, Starvation therapy, Chemodynamic therapy, Multifunctional cascade nanoreactor

## Abstract

The rapid growth of tumors relies heavily on a continuous supply of essential nutrients, including glucose and copper. Disrupting the nutrient supply to tumors has become an increasingly focal point in tumor therapy. However, solely blocking the energy supply typically only hinders further tumor growth and may not effectively eliminate existing tumor cells. Herein, a multifunctional cascade nanoreactor (HPP/TPEN@GC) endowed with N, N, N′, N′-tetrakis(2-pyridinylmethyl)-1,2-ethanediamine (TPEN, a copper chelator) and glucose oxidase (GOx) is designed to disrupt both glycolysis and mitochondrial metabolism, which further induce cascade chemodynamic therapy (CDT). HPP/TPEN@GC can react with endogenous copper and glucose, thereby reducing their availability. The absence of copper prevents proper assembly and function of mitochondrial complex IV (CIV), hindering mitochondrial metabolism; the lack of glucose cuts off glycolysis and leads to a tumor specific starvation. Meanwhile, the reactions catalyzed by HPP/TPEN@GC contribute to the generation of Fenton-like catalysts and hydrogen peroxide (H_2_O_2_), which can further react to produce highly toxic hydroxyl radical (·OH) for CDT. Taken together, the multifunctional cascade nanoreactor reduces the availability of endogenous copper and glucose, and further takes advantage of them to generate ·OH for cascade starvation-chemodynamic therapy. Collectively, this work represents a distinctive therapeutic paradigm to harness endogenous copper and glucose, which should inspire further studies to take full advantage of endogenous nutrients to combat various diseases, including tumors.

## Highlights

• The strategy of reducing the availability of endogenous copper and glucose is proposed for cancer therapy.
• A multifunctional cascade nanoreactor is designed to disrupt glucose metabolism and break the redox homeostasis.
• TPEN reduces the availability of copper, which hinders mitochondrial respiration and generates copper complexes.
• Glucose oxidase reduces the availability of glucose, which inhibits glycolysis and induces the generation of H_2_O_2_.
• The copper complexes catalyze the conversion of H_2_O_2_ into highly toxic ROS to kill tumor cells.

## Graphical abstract

**Figure:**
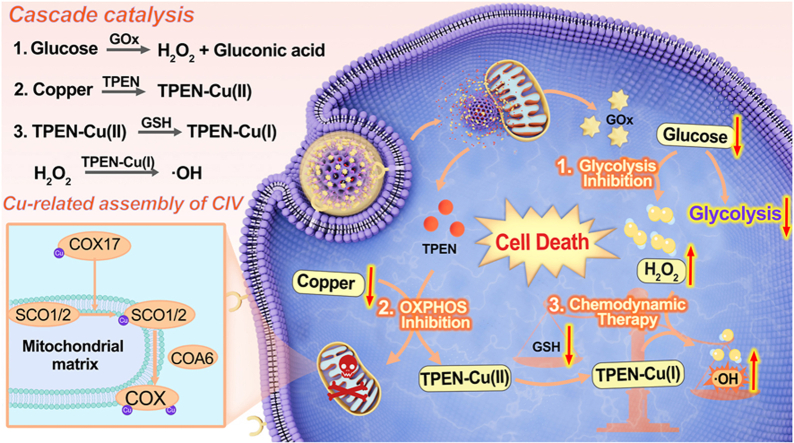

## 1 Introduction

As an indispensable trace element, copper (Cu) has attracted wide attention in tumor therapy owing to its unique physicochemical properties as well as its crucial physiological functions [1,2]. Cu-based nanotherapeutics have been extensively studied and developed, including copper nanoparticles [3,4], copper oxides [[5], [6], [7]], copper peroxides [8,9], copper chalcogenides and Cu-related metal-organic frameworks [10]. However, current research has mostly focused on supplying exogenous copper to kill tumor cells and inhibit tumor growth [[11], [12], [13], [14]]. It should be noted that endogenous copper plays an important role in the occurrence, development and metastasis of tumors. For example, copper can provoke the canonical RAF-MEK-ERK pathway and regulate various pro-angiogenic factors to promote cell proliferation and angiogenesis, respectively; copper is a crucial cofactor of Cu-Zn superoxide dismutase, which is important for maintaining cellular redox homeostasis; increased copper levels also improve the expression of PD-L1 to suppress the immune response [15]. Specifically, the copper concentration in tumors is higher than that of normal tissues [16]. Therefore, designing feasible therapeutic agents based on endogenous copper is an alternative promising strategy for tumor treatment [[17], [18], [19]].

The Cu-dependent cytochrome *c* oxidase, also known as mitochondrial CIV, is the terminal constituent of the mitochondrial electron transport chain, and plays a crucial role in maintaining the energy supply and biosynthesis in cells. Copper is an essential cofactor for cytochrome *c* oxidase and the absence of copper leads to the rapid degradation of the subunits, thereby hindering the assembly and function of CIV [20,21]. Therefore, limiting the availability of copper by copper chelators is potential to interfere with the physiological function of CIV and disrupt the mitochondrial oxidative phosphorylation (OXPHOS) [22]. However, tumor cells can obtain energy not only through OXPHOS, but also through aerobic glycolysis, which is commonly known as the Warburg effect [23,24]. In addition, tumor cells can switch between the two metabolic pathways when confronted with metabolic challenges [23]. As a result, inhibition of the OXPHOS alone cannot completely cut off the energy supply to the tumor.

To solve the problem, the strategy targeting both OXPHOS and aerobic glycolysis is highly demanded. Tumor cells favor aerobic glycolysis, which means they rely on glycolysis even in the presence of oxygen, unlike normal cells [25,26]. This metabolic characteristic requires tumor cells to consume significantly more glucose, approximately 10 times more than normal cells, to sustain their energy supply and synthesis of biomolecules [27]. Thus, cutting off the glucose supply can effectively hinder the energy supply to the tumor [28]. One potential strategy is to consume endogenous glucose to reduce its availability. GOx, an enzyme that catalyzes the oxidation of glucose to produce gluconic acid and H_2_O_2_, appears to be an ideal candidate [29,30].

Surprisingly, the utilization of GOx in conjunction with copper chelators can further induce enhanced CDT. Although both H_2_O_2_ and ·OH are classified as ROS, H_2_O_2_ exhibits lower cytotoxicity and limited efficacy in killing tumor cells. ·OH is recognized as the most biotoxic species within the ROS family and holds promise for tumor therapy due to its potent cytotoxicity [31]. Therefore, it is a feasible therapeutic strategy to convert overexpressed H_2_O_2_ at tumor sites into highly cytotoxic ·OH [32,33]. Under reductive physiological conditions, the complex formed by chelating copper can serve as a catalyst for the Fenton-like reaction to induce CDT. In addition, GOx-catalyzed glucose oxidation promotes the accumulation of H_2_O_2_, which compensates for its insufficient concentration, thereby enhancing the efficacy of CDT [34,35].

**Scheme 1:**
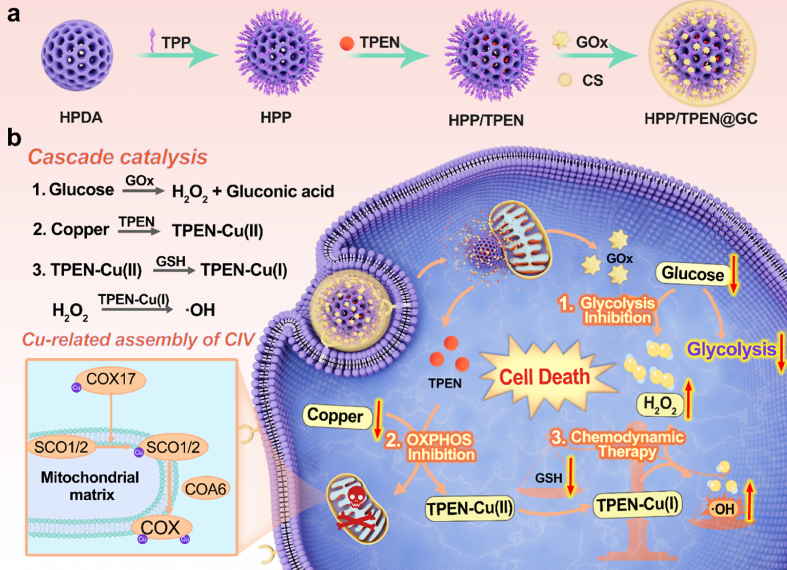
Schematic illustration of a) the preparation of HPP/TPEN@GC and b) the therapeutic mechanisms: (1) the glucose oxidation catalyzed by GOx to deprive glucose and induce H_2_O_2_ accumulation for glucose starvation and enhanced CDT; (2) the copper depletion to interrupt the assembly of CIV and generate TPEN-Cu(II) for OXPHOS inhibition and further CDT; (3) the Fenton-like reaction in the presence of TPEN-Cu(I) and H_2_O_2_ for CDT.

Herein, a multifunctional cascade nanoreactor (HPP/TPEN@GC) endowed with TPEN and GOx was constructed to reduce the availability of endogenous copper and glucose, inhibiting glycolysis and OXPHOS for cascade starvation therapy and CDT (Scheme 1). To achieve this goal, hollow polydopamine (HPDA) nanoparticles were chosen as the nanocarrier to co-load TPEN and GOx. Meanwhile, chondroitin sulfate (CS) was coated on the surface of the nanoparticles to avoid GOx inactivation in blood circulation and confer tumor-targeting ability [36,37]. After intravenous injection, HPP/TPEN@GC would accumulate at the tumor site through the interaction between CS and overexpressed CD44 receptor [36,37]. Then GOx could catalyze the oxidation of glucose, inhibiting the glycolysis as well as promoting the accumulation of H_2_O_2_. Concurrently, the released TPEN could chelate endogenous copper and disrupt OXPHOS in cancer cells. In addition, the generated copper complex TPEN-Cu(I) would function as a catalyst of Fenton-like reaction, which could convert both endogenous H_2_O_2_ and generated H_2_O_2_ into ·OH for enhanced CDT. Along with the cascade starvation therapy and CDT, HPP/TPEN@GC demonstrated a potent therapeutic outcome both in vitro and in vivo. This study provided an alternative promising therapeutic strategy to disrupt the glycolysis and mitochondrial metabolism through reducing the availability of endogenous glucose and copper for the treatment of triple-negative breast cancer.

## 2 Methods

### 2.1 Materials

Tetraethyl orthosilicate (TEOS) and chondroitin sulfate (CS) were purchased from Macklin (Shanghai, China). Ammonium hydroxide (NH_3_⋅H_2_O, 25 %–28 %, w/w), tris (hydroxymethyl) aminomethane, dopamine hydrochloride (DA⋅ HCl) and hydrofluoric acid (HF) were obtained from Sigma Aldrich (Shanghai, China). Anhydrous ethanol, 3-carboxypropyltriphenylphosphonium bromide (TPP-COOH), 1-(3-diaminopropyl)-3ethylcarbodiimide hydrochloride (EDC), N-hydroxysuccinimide (NHS) and methylene blue (MB) were acquired from Adamas (Shanghai, China). N, N, N′, N′-tetrakis(2-pyridinylmethyl)-1,2-ethanediamine (TPEN) and Rhodamine B hydrazide (RBH) were acquired from Bide Pharmatech Ltd (Shanghai, China). Glucose oxidase (GOx) was obtained from Medchemexpress (Shanghai, China). 2′,7′-dichlorofluorescin diacetate (DCFH-DA), BCA protein assay kit, Glutathione (GSH) assay kit and mitochondrial membrane potential (MMP) assay kit were obtained from Beyotime (Shanghai, China). Hoechst 33342, cell viability test kit and CCK-8 assay kit were purchased from KeyGEN BioTECH (Nanjing, China). Annexin V-FITC/PI staining kit, Goat anti-Rabbit IgG/FITC and H&E staining kit were obtained from Solarbio (Beijing, China). All reagents were analytical without further purification.

### 2.2 Synthesis of monodisperse solid SiO_2_ nanoparticles

SiO_2_ nanoparticles were synthesized as previously reported [38]. Briefly, 28 mL ethanol, 4 mL water and 1 mL NH_3_·H_2_O were mixed and the mixture was heated to 50 °C. Next, 1 mL TEOS was added dropwise and the reaction continued for 2 h with constant stirring. Solid SiO_2_ nanoparticles were obtained by centrifugation and washed twice with ethanol and deionized water.

### 2.3 Synthesis of hollow mesoporous PDA (HMPDA) nanoparticles

The as-prepared SiO_2_ nanoparticles were dispersed in Tris buffer (10 mM, pH = 8.5) under ultrasound [39]. Next, 150 mg DA⋅HCl was dissolved in water and the obtained solution was poured into the reaction mixture. After stirring overnight at room temperature, SiO_2_@PDA nanoparticles were collected by centrifugation (10000 rpm, 10 min) and washed with water. Subsequently, the SiO_2_@PDA nanoparticles were dispersed in 1.67 wt% HF solution and kept overnight in a static state. The HMPDA nanoparticles were harvested by centrifugation (12000 rpm, 10 min) and washed with water twice.

### 2.4 Synthesis of TPP modified HMPDA nanoparticles (HPP NPs)

TPP-COOH was linked to the surface of HMPDA via the EDC/NHS reaction. In brief, TPP-COOH (12 mg), EDC (21.6 mg) and NHS (14.4 mg) were mixed with DMSO in the dark to activate carboxyl groups. After stirring for 2 h, HMPDA solution was added into the above mixture and the reaction continued under gentle stirring overnight. The HPP NPs were separated and washed with water twice.

### 2.5 Synthesis of TPEN-loaded HPP nanoparticles (HPP/TPEN NPs)

The obtained HPP NPs were dispersed in a solution of TPEN in ethanol and the mixture was stirred at room temperature for 24 h [40]. The resulting HPP/TPEN NPs were collected by centrifugation, purified with water and dispersed in deionized water for further use.

### 2.6 Synthesis of HPP/TPEN@GC NPs

GOx (1 mg) and CS (2 mg) were dissolved in deionized water and the as-prepared HPP/TPEN solution was added to the mixture with constant stirring at room temperature. After 24 h, the reaction mixture was centrifuged and washed to obtain the final HPP/TPEN@GC NPs.

### 2.7 Characterizations

Transmission electron microscopic (TEM) images were acquired on TEOL JEM-2100 transmission electron microscope. The hydrodynamic sizes and zeta potentials were conducted on an Anton Paar Litesizer 500 instrument at 25 °C. Fourier transform infrared (FT-IR) spectra were recorded on a Nicolet iS10 spectrometer. The X-ray photoelectron spectra (XPS) were measured by a Thermo ESCALAB 250XI electron spectrometer. Ultraviolet–visible (UV–vis) absorption spectra were taken on a Hitachi U-3900 spectroscopy. The fluorescence spectra were collected on a Hitachi F-7000 spectrofluorophotometer. The cellular uptake behavior was verified by a ZEISS Axio Vert A1 fluorescence microscopy and a CytoFLEX LX flow cytometry.

### 2.8 Response of RBH to copper

RBH was selected as a copper ion fluorescent probe. To verify the selectivity of RBH for copper ions, RBH (2 μM) was mixed with a variety of metal ions. After a few minutes of incubation, the fluorescence spectra of the mixture were collected at 488 nm of excitation. To verify the copper chelating ability of TPEN, RBH (2 μM) was mixed with different concentrations of copper ion solutions (0 μM, 1 μM, 2 μM, 5 μM and 10 μM) with or without TPEN. After a few minutes of incubation, the fluorescence spectra of the mixture were collected at 488 nm of excitation.

### 2.9 Detection of dissolved oxygen

Portable dissolved oxygen meter (JPB-607A, INESA) with a DO-957 dissolved oxygen electrode was used to monitor the real-time change of dissolved oxygen. Briefly, HPP/TPEN@GC (10 μg/mL, 10 mL) was incubated with 10 mM glucose solution under constant stirring and the electrode was immersed in the solution to monitor the content of dissolved oxygen.

### 2.10 Detection of H_2_O_2_ generation

Ammonium titanyl oxalate was utilized as a sensor to detect the generation of H_2_O_2_ in vitro. In brief, HPP/TPEN@GC (20 μg/mL, 5 mL) was incubated with 10 mM glucose solution and after various time intervals (0 h, 0.25 h, 0.5 h, 1 h, 1.5 h, 2 h, 3 h and 4 h) the supernatant was obtained by centrifugation. The solution of ammonium titanyl oxalate (10 mM, 50 μL) was added to react for a few minutes. The UV–vis absorption spectra were collected and the concentrations of H_2_O_2_ were calculated according to the standard curve. Moreover, HPP/TPEN@GC (20 μg/mL, 5 mL) was also incubated with various concentrations of glucose (0 mM, 0.1 mM, 0.2 mM, 0.5 mM, 1 mM, 2 mM, 5 mM, 10 mM and 15 mM) and the generated H_2_O_2_ was quantified by the same method.

### 2.11 Detection of ·OH

The ability of TPEN-Cu(I) to generate ∙OH was determined using MB as a sensor. TPEN-Cu(I) was obtained by incubating TPEN with copper ions and GSH at 37 °C for 1h. Next, the obtained TPEN-Cu(I) was mixed different concentrations of H_2_O_2_ and meanwhile MB was added to detect the generation of ∙OH. After incubation at 37 °C for some time, the UV–vis spectra were collected to indicate the generation of •OH.

### 2.12 Drug release

3 mg HPP/TPEN@GC was dispersed in 1 mL phosphate buffered solution (pH = 7.4) and incubated at 37 °C for 48 h. At designed time intervals, the solution was centrifuged to collect 0.8 mL supernatant and the same volume of fresh phosphate buffered solution was supplemented. The content of TPEN in the collected supernatant was quantified according to the UV–vis absorption and the cumulative release was calculated.

### 2.13 Cellular uptake behavior

For concentration-dependent uptake experiments, EMT-6 cells were cultured in 12-well plates overnight and exposed to Rhodamine B (RhB)-labeled HPP/TPEN@GC (0, 2.5, 5 and 10 μg/mL) for 4 h. Subsequently, these EMT-6 cells were observed under fluorescence microscopy and assessed by flow cytometry. For time-dependent uptake experiments, EMT-6 cells were exposed to 10 μg/mL RhB-labeled HPP/TPEN@GC for 0–6 h. Then, the experiments were performed the same as above.

### 2.14 Cytotoxicity assay

CCK-8 assay kit was used to assess cytotoxicity. 1 × 10^4^ EMT-6 cells per well were seeded in 96-well plates overnight. Then, these cells were cocultured with HPP@C, HPP/TPEN@C, HPP@GC and HPP/TPEN@GC at different concentrations with or without glucose for 24 h. After that, the media of each well was replaced by 100 μL of CCK-8 solution (containing 10 % CCK-8 reagent) for 2 h. The cell viability was evaluated according to 450 nm absorbance intensity.

### 2.15 Live/dead cell staining assay

2 × 10^5^ EMT-6 cells per well were seeded in 12-well plates. After overnight incubation, the cells were exposed to HPP@C, HPP/TPEN@C, HPP@GC and HPP/TPEN@GC (10 μg/mL) for 24 h. Then, the cells were co-stained with Calcein-AM and PI solutions for 30 min in the dark. Finally, the cells were washed with PBS and imaged by fluorescence microscopy.

### 2.16 Apoptosis assay

Annexin V-FITC/PI staining kit was used to quantitative cell apoptosis of HPP/TPEN@GC. 2 × 10^5^ EMT-6 cells per well were seeded in 12-well plates overnight. After that, the cells were treated with HPP@C, HPP/TPEN@C, HPP@GC and HPP/TPEN@GC (10 μg/mL) for 24 h. Next, the cells were co-stained with PI and Annexin V-FITC for 15 min and then evaluated by flow cytometry.

### 2.17 Intracellular ROS generation

DCFH-DA was used to detect intracellular ROS generation. 2 × 10^5^ EMT-6 cells per well were plated in 12-well plates overnight. Afterwards, the cells were treated with HPP@C, HPP/TPEN@C, HPP@GC and HPP/TPEN@GC for 4 h. After incubation with DCFH-DA probe for 20 min, the cells were recorded by fluorescence microscopy and flow cytometry.

### 2.18 Intracellular GSH assay

Total GSH was quantified using GSH assay kit. EMT-6 cells were seeded in 6-well plates overnight and then treated with HPP@C, HPP/TPEN@C, HPP@GC and HPP/TPEN@GC for 4 h. Finally, the assay was conducted according to the manufacturer's instructions.

### 2.19 Detection of CRT expression

Surface expression of CRT was detected by flow cytometry. EMT-6 cells were collected and incubated with CRT primary antibody for 1 h at 4 °C. After blocking, the samples were incubated with secondary antibody for 1 h. The fluorescence intensity of each sample was analyzed.

### 2.20 Western blotting

After different treatments, total protein of EMT-6 cells was collected. Subsequently, the proteins were subjected to 15 % SDS-PAGE electrophoresis, transferred to PVDF membranes and blocked according to standard protocols. The membranes were then incubated with MTCO1 antibody (1:1000) and β-Tubulin (1:5000) at 4 °C overnight. After secondary antibody incubation, the protein blots were detected by autoradiography.

### 2.21 MMP assay

The MMP was measured by JC-1 dye based MMP assay kit. After different treatments, EMT-6 cells were washed and incubated with JC-1 solution at 37 °C for 20 min. Consequently, the cells were washed and recorded by flow cytometry. MMP is calculated as red/green fluorescence intensity ratio.

### 2.22 Animal model

5-week-old female BALB/c mice were purchased from Shanghai Laboratory Animal Center (Shanghai, China). The tumor xenograft model was established by injecting 5 × 10^5^ EMT-6 cells into the right mammary fat pad of the mice. All animal procedures conformed to the Guide for the Care and Use of Laboratory Animals (ethical approval number: TJBB00723105).

### 2.23 In vivo biodistribution of HPP/TPEN@GC

When EMT-6 tumors reached a volume of 200–300 mm^3^, mice were randomly divided into three groups and were intravenously injected with ICG, ICG-HPP/TPEN@G and ICG-HPP/TPEN@GC (ICG, 0.5 mg/kg), respectively. Then, the mice were recorded at different time points during 0–48 h by an animal imaging system. Finally, the major organs and tumors were harvested and imaged for further tissue distribution study.

### 2.24 In vivo antitumor therapy

When tumor volume reached approximately 50 mm^3^, EMT-6 tumor bearing mice were randomly divided into five groups (n = 5) for the treatments with PBS, HPP@C, HPP/TPEN@C, HPP@GC and HPP/TPEN@GC (25 mg/kg, 100 μL) every three days. Along with administration, mice weight and tumor volume (tumor volume = 0.5 × length × width^2^) were measured. Three days after the last administration, all mice were sacrificed and then the tumors were excised and weighed. Moreover, tumors, main organs and blood samples were collected for histological analysis and biochemical analysis according to standard protocols.

### 2.25 In vivo immune cells population detection

At the end of treatments, tumor tissues and spleens were dispersed into single-cell suspensions. Followed by splitting red blood cells by red blood cell lysis buffer, the samples were stained with fluorescence conjugated antibodies according to the manufacturer's instructions. The samples were washed, resuspended using cell staining buffer, and analyzed by flow cytometry.

### 2.26 Statistical analysis

All data were expressed as mean ± SD. Student *t*-test was performed to compare the differences between groups. The significant difference between two groups was expressed as ∗p < 0.05, ∗∗p < 0.01, ∗∗∗p < 0.001 and ∗∗∗∗p < 0.0001.

## 3 Results and discussion

### 3.1 Bioinformatics analyses of Cu-related genes

**Fig. 1:**
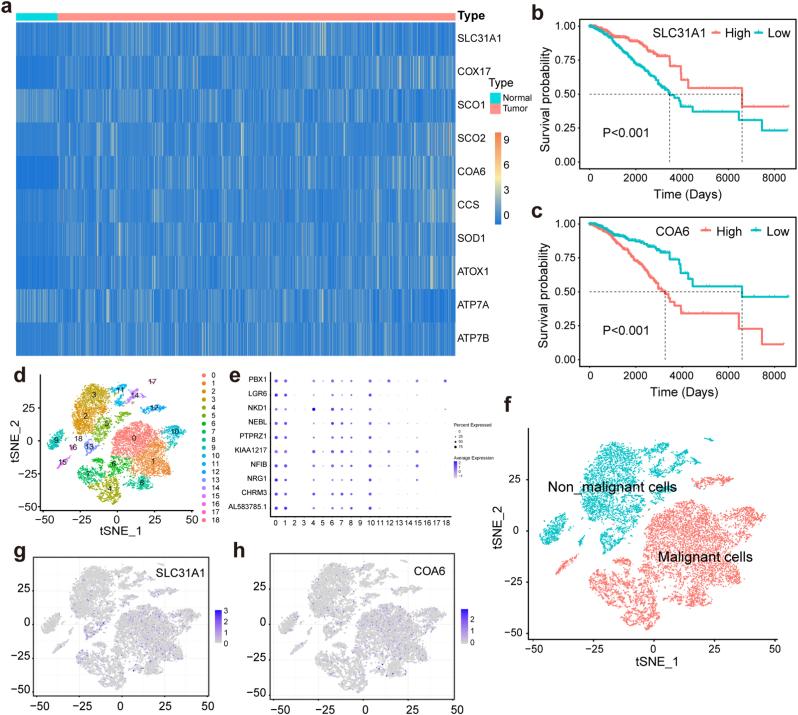
The upregulated demand for copper in patients with triple-negative breast cancer. a) Heatmap of genes associated with copper uptake and transport in the TCGA-BRCA dataset. b) Effects of SLC31A1 expression on survival time of patients with BRCA. c) Effects of COA6 expression on survival time of patients with BRCA. d) t-SNE (t-distributed Stochastic Neighbor Embedding) plot showing the cellular subpopulation in all the clusters of GSE252175. e) Bubble plots showing the expression levels of tumor cell marker genes in different cell subpopulations. f) t-SNE plot showing the malignant and non-malignant cells after cell annotation. g) The expression level of SLC31A1 in different cell subpopulations. h) The expression level of COA6 in different cell subpopulations.

Copper is closely related to the occurrence, development and metastasis of tumors. Endogenous copper is not only involved in the regulation of various signalling pathways in vivo, but also a cofactor of several important proteins [2]. Due to the rapid proliferation, tumors tend to have a higher copper demand than normal tissues. To compare the expression levels of genes associated with copper uptake and transport in healthy tissues and breast cancer, the transcriptome data of breast cancer patients were obtained from The Cancer Genome Atlas database (TCGA), breast cancer (BRCA) dataset. This analysis included 1118 tumor tissue samples and 113 normal tissue samples. Genes associated with copper uptake and transport were screened from the transcriptome data and their differences were analyzed. As shown in Fig. 1a and Table S1, most genes related to copper uptake and transport were overexpressed in tumor tissues, indicating an increased demand for copper in these tissues. Notably, SLC31A1 (Solute Carrier Family 31 Member 1, also known as copper transporter 1) is primarily responsible for the intracellular uptake of copper, while cytochrome *c* oxidase 17 (COX17), synthesis of cytochrome oxidase 1 (SCO1), SCO2, and cytochrome *c* oxidase assembly factor 6 (COA6) are closely involved in the targeted insertion of copper into metalloprotein and the assembly of CIV. SLC31A1 and COA6 were selected as two representative genes for subsequent analysis. To investigate the correlation between the expression of selected genes and survival time in tumor patients, the patients were classified into high and low expression groups based on the levels of SLC31A1 and COA6, followed by a subsequent analysis of their survival durations. As demonstrated in Fig. 1b and c, patients with high expression of SLC31A1 experienced shorter survival times, and similar results were observed for COA6. These findings suggest that reducing the availability of endogenous copper could be a promising therapeutic target for tumors.

To further confirm the high copper demand in tumor cells rather than other cell types in the tumor tissue, the expression levels of SLC31A1 and COA6 were further analyzed at the single-cell level in breast cancer tissue. In the GSE252175 dataset, all tumor cells were classified into 19 clusters after dimensionality reduction and cluster analysis (Fig. 1d). After checking the expression levels of tumor cell marker genes in each subgroup (Fig. 1e), the 9 subgroups of 0, 1, 4, 6, 7, 8, 10, 12, and 15 were designated as malignant cells, and the other cell subgroups were designated as non-malignant cells (Fig. 1f) [41]. Compared to non-malignant cells, the expression levels of SLC31A1 and COA6 were both higher in malignant cells (Fig. 1g and h), which further indicated the increased copper demand in tumor cells. Thus, TPEN (a kind of copper chelator) was chosen to reduce the availability of endogenous copper for cancer therapy.

Additionally, the expression of glucose transporter type 1 (GLUT1) was positively correlated with the expression of both SLC31A1 and COA6 (Fig. S1). The survival analysis also demonstrated that the high expression of GLUT1 may result in a shorter survival time (Fig. S2). Herein, a multifunctional cascade nanoreactor (HPP/TPEN@GC) endowed with TPEN and GOx was designed to reduce the availability of endogenous copper and glucose simultaneously.

### 3.2 Synthesis and characterizations of HPP/TPEN@GC

**Fig. 2:**
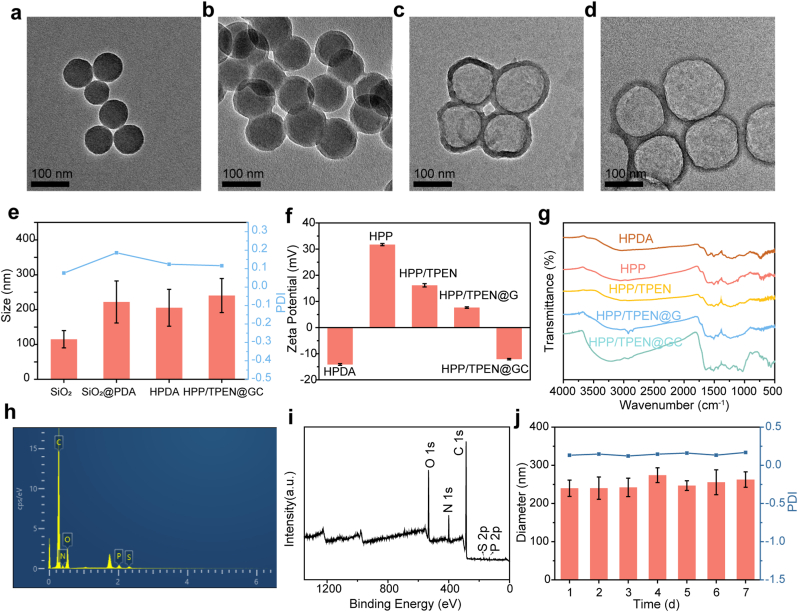
Synthesis and characterizations of HPP/TPEN@GC. TEM images of a) SiO_2_, b) SiO_2_@PDA, c) HPDA and d) HPP/TPEN@GC. e) Hydrodynamic diameters and PDI of SiO_2_, SiO_2_@PDA, HPDA and HPP/TPEN@GC. f) Zeta potentials and g) FT-IR spectra of HPDA, HPP, HPP/TPEN, HPP/TPEN@G and HPP/TPEN@GC. h) EDS elemental analysis of HPP/TPEN@GC. i) XPS spectrum of HPP/TPEN@GC. j) The stability of HPP/TPEN@GC.

The detailed synthetic process and in vivo application of HPP/TPEN@GC are displayed in Scheme 1. HPDA nanoparticles were first synthesized through the template-eliminated methods and monodispersed SiO_2_ nanoparticles were used as the sacrificial template [42]. After the success synthesis of SiO_2_ nanoparticles, the PDA shell was coated on their surface through in situ oxidative polymerization under alkaline conditions [39]. The resulted SiO_2_@PDA nanoparticles were treated with hydrofluoric acid solution to remove the template core and obtain HPDA nanoparticles. As shown in Fig. 2a and e, the prepared SiO_2_ nanoparticles exhibited spherical morphologies and the hydrodynamic diameter was 114.85 ± 24.81 nm with a polydispersity index (PDI) of 0.076. After coated with PDA shell, the hydrodynamic diameter increased to 221.91 ± 60.32 nm, which indicated that SiO_2_@PDA nanoparticles were successfully fabricated. Transmission electron microscopy (TEM) images showed the uniform yolk–shell structure of SiO_2_@PDA nanoparticles and the thickness of the PDA shell was ∼10 nm (Fig. 2b and Fig. S3). Subsequently, the SiO_2_ core was etched by hydrofluoric acid solution and HPDA nanoparticles were successfully obtained. The TEM image of HPDA nanoparticles clearly showed the hollow structures (Fig. 2c) and dynamic light scattering (DLS) measurements demonstrated that the hydrodynamic diameter of HPDA was 205.30 ± 52.96 nm, which was slightly smaller than that of SiO_2_@PDA (Fig. 2e). This was most likely due to the partial degradation of the PDA shell caused by the acidic environment during the etching of SiO_2_. As displayed in Fig. 2c and Fig. S4 , HPDA exhibited unique hollow structure and good stability, suggesting it was a good candidate as a drug nanocarrier. Conveniently, HPDA exhibited a concentration-dependent UV–Vis absorption, which allowed it to be quantified according to a standard curve (Fig. S6).

To enhance the accumulation of copper chelators in mitochondria, triphenylphosphine (TPP) was conjugated to the surface of HPDA via an amide reaction [43,44]. After the modification of the positive TPP ligand, the zeta potential of HPP increased from −14.11 mV to +31.71 mV (Fig. 2f). In addition, the Fourier-transform infrared (FT-IR) spectra were also obtained to confirm the successful modification of TPP (Fig. 2g and Fig. S5a). The emerging bands at around 1438 cm^−1^ was consist with the absorption band of TPP, which was contributed to the aromatic ring skeleton stretching vibration [45]. TPEN, the copper chelator, was then loaded into the hollow mesoporous structure of HPP to obtain HPP/TPEN [40]. The UV–vis absorption spectra were collected before and after TPEN loading and the loading efficiency was calculated to be 10.4 wt% ([Fig. S7 and [Fig. S8). Furthermore, GOx was absorbed on the surface of HPP/TPEN due to the electrostatic interaction between the positive HPP/TPEN and the negative GOx [46,47]. The new absorption bands at 2854 cm^−1^ and 2925 cm^−1^ further confirmed the presence of GOx in the nanoreactor (Fig. 2g and Fig. S5c). As shown in Fig. 2f, the zeta potential changed from +16.17 mV to +7.66 mV after GOx loading. The loading efficiency of GOx in the nanocarrier was quantified with the BCA Protein Assay Kit and measured to be 5.3 wt%. Furthermore, CS was coated on HPP/TPEN@G to shield the positive surface and avoid GOx inactivation in blood circulation. After the coating of CS, the zeta potential of HPP/TPEN@GC correspondingly converted to −12.08 mV (Fig. 2f). As displayed in Fig. 2g and Fig. S5d, the new bands at around 1030 cm^−1^ and 1246 cm^−1^ in the FT-IR spectrum of HPP/TPEN@GC indicated the successful modification of CS. Energy dispersive spectrometry (EDS) spectrum confirmed the existence of C, N, O, P, and S elements, illustrating the successful modification of TPP and CS with the corresponding P and S elements, respectively (Fig. 2h). XPS analysis also displayed the presence of C, N, O, P, and S elements (Fig. 2i and Fig. S9), which was consistent with the results of EDS analysis. The obtained HPP/TPEN@GC was 240.37 ± 48.99 nm with a PDI of 0.116 (Fig. 2e) and demonstrated spherical morphologies with a distinct polymer layer (Fig. 2d). As expected, HPP/TPEN@GC maintained a uniform size and good dispersion in 7 days (Fig. 2j).

In addition, the release of TPEN was studied by incubating HPP/TPEN@GC in PBS at 37 °C for 48 h. As shown in Fig. S10, TPEN was gradually released during the experimental process and the cumulative release reached up to 33.6 % at 48 h. The sustained release of TPEN was effectively realized and there was still a persistent release of TPEN after 24 h. This might be due to the presence of hydrogen bond and π-π stacking interaction between TPEN and HPP, which brought down the drug release rate.

### 3.3 Cascade catalytic performance of HPP/TPEN@GC

**Fig. 3:**
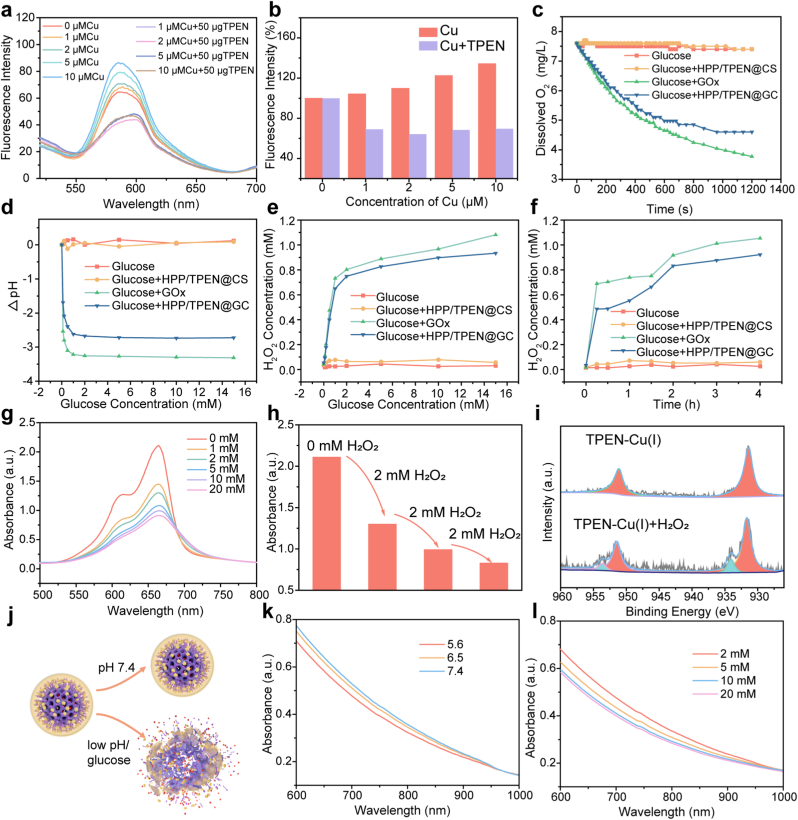
*In vitro* catalytic performance of HPP/TPEN@GC. a) Fluorescence spectra and b) the fluorescence intensity of RBH after mixed with different concentrations of copper ions with or without TPEN. c) Dissolved oxygen in the glucose solution after treated with different nanoparticles. d) The pH changes and e) H_2_O_2_ generation after incubated with different concentrations of glucose. f) H_2_O_2_ generation at designed time intervals. g) UV–vis spectra of MB to indicate the capability of TPEN-Cu(I) to produce ·OH after incubated with different concentrations of H_2_O_2_. h) UV–vis absorbance of the mixed solution containing TPEN-Cu(I) and MB after the additions of H_2_O_2_. i) XPS spectra of TPEN-Cu(I) in the absence and presence of H_2_O_2_. j) Schematic representation of the biodegradation process under different conditions. k) UV–vis spectra of HPP/TPEN@GC after incubated at different pH. l) UV–vis spectra of HPP/TPEN@GC after incubated with different concentrations of glucose.

TPEN and its analogues can serve as copper chelators to deprive copper [40,48]. To study the chelating ability of TPEN to copper ions more clearly, Rhodamine B hydrazide (RBH) was utilized as the copper-specific fluorescence probe, which was highly selective to divalent copper ions (Fig. S11) [49]. As displayed in Fig. 3a and b, after incubation with copper ions, the fluorescence intensity of RBH exhibited a noticeable enhancement. Furthermore, as the concentration of copper ions increased, the fluorescence intensity showed a corresponding gradual enhancement. However, the addition of TPEN resulted in a distinct difference. The fluorescence intensity of RBH in the TPEN-containing groups ceased to rise as the concentration of copper ions increased. Instead, it remained consistently at a relatively low level. This observation suggested that the concentration of copper ions in the solution is minimal, thereby demonstrating TPEN's exceptional ability to chelate copper ions even at low concentrations.

Subsequently, the catalytic ability of GOx was investigated. In the presence of oxygen, GOx could catalyze the oxidation of glucose to generate gluconic acid and H_2_O_2_ [50]. First, the dissolved oxygen was detected to verify the activity of GOx in HPP/TPEN@GC. As shown in Fig. 3c, upon the addition of HPP/TPEN@GC to the glucose solution, a rapid decline in dissolved oxygen was observed, which was similar to the trend in the glucose + GOx group. However, there was no significant change in dissolved oxygen levels within the two control groups (pure glucose solution and HPP/TPEN@C-containing solution). This finding demonstrated that GOx retained a robust catalytic ability in this nanoreactor. In parallel, the variation of pH in the solution was also examined over time after incubating HPP/TPEN@GC with glucose. It was observed that the pH of the solution gradually decreased, aligning with the changes in dissolved oxygen levels (Fig. S12). As shown in Fig. 3d, the pH of the solution decreased as the concentration of glucose increased, indicating a corresponding rise in the production of gluconic acid. The decline in pH is advantageous for promoting the biodegradation of HPDA and minimizing potential long-term biological toxicity. More importantly, the generation of H_2_O_2_ was investigated over time and in relation to varying glucose concentrations. The produced H_2_O_2_ was quantified according to the standard curve of UV–Vis absorption (Fig. S13). With the extension of time, there was a gradual increase in the amount of H_2_O_2_ generated, indicating that GOx exhibited a continuous ability to consume glucose and generate H_2_O_2_ (Fig. 3f). Additionally, as the glucose concentration increased, the production of H_2_O_2_ initially showed a rapid increase and eventually reached a plateau, suggesting that the maximum catalytic speed of GOx might have reached (Fig. 3e).

Next, the ·OH generation ability was evaluated using methylene blue (MB) as an indicator [51]. TPEN was first introduced to Cu^2+^-containing solution to generate TPEN-Cu(II). To simulate the bioreductive microenvironment, glutathione (GSH) was added to the mixture to facilitate the reduction of the copper complex, resulting in the formation of TPEN-Cu(I). As shown in Fig. 3i, following the treatment with GSH, nearly all copper ions existed in the form of Cu(I). After that, the generated TPEN-Cu(I) was incubated with different concentrations of H_2_O_2_ to study its catalytic ability of producing ·OH. As displayed in Fig. 3g, the absorption intensity of MB at 662 nm decreased with the increase of H_2_O_2_ concentration, suggesting that the amount of ·OH generated increased gradually. To further investigate the sustainable use of TPEN-Cu(I) as a catalyst, H_2_O_2_ was added to the MB-containing TPEN-Cu(I) solution in batches and the UV–vis absorption spectra were collected. Following each addition of H_2_O_2_, the UV–vis absorption of MB displayed a noticeable reduction, but the pace of decline markedly decelerated (Fig. 3h). XPS analysis revealed that 11.39 % of TPEN-Cu(I) transformed into TPEN-Cu(II) after treated by H_2_O_2_, potentially due to oxidation by atmospheric oxygen (Fig. 3i). Based on these findings, TPEN-Cu(I) exhibited the potential to catalyze the generation of ·OH, but it underwent a gradual decline in catalytic capacity due to the oxidation of copper. Moreover, the ·OH production ability of HPP/TPEN@GC was further investigated. In the presence of H_2_O_2_, most of the MB was degraded, indicating the generation of ·OH (Fig. S14a). With the increase of H_2_O_2_ concentration, the amount of ·OH produced also increased gradually, which proved that H_2_O_2_ supplementation was an effective way to enhance CDT (Fig. S14b).

### 3.4 The degradation of HPP/TPEN@GC

It was supposed that HPP/TPEN@GC could be degraded under acidic conditions or after incubation with glucose (Fig. 3j). To verify this property, the degradation of the nanoparticles was investigated by collecting their UV–Vis absorption spectra after incubation under different conditions, as the decrease in absorption could indicate the degradation of the nanoparticles [52]. As shown in Fig. S15a, HPDA could be degraded under acidic conditions, and the lower the pH, the more degradation. HPP/TPEN@CS and HPP/TPEN@GC showed similar results after incubation at different pH conditions (Fig. 3k and Fig. S15b). Additionally, HPP/TPEN@CS and HPP/TPEN@GC were respectively incubated with different concentrations of glucose to evaluate their degradation. There was almost no change in the UV–Vis absorption of HPP/TPEN@CS (Fig. S16). In contrast, the absorption of HPP/TPEN@GC obviously decreased, which was contributed to the accumulation of gluconic acid produced by GOx-catalyzed glucose oxidation (Fig. 3l). These results indicated that HPP/TPEN@GC could be degraded in the acidic tumor microenvironment and the degradation could be accelerated by the accumulated gluconic acid.

### 3.5 Cellular uptake efficiency and synergistic efficacy

**Fig. 4:**
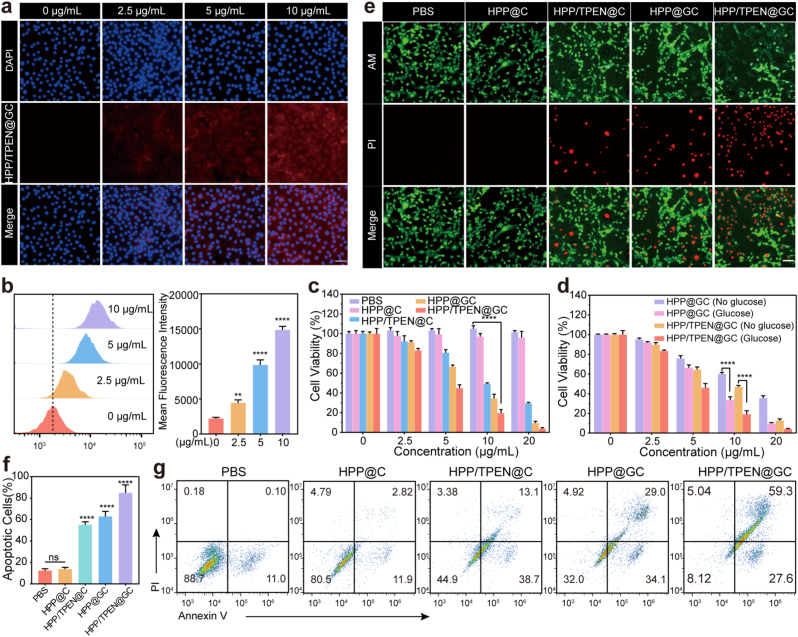
Cellular uptake efficiency and synergistic efficacy of HPP/TPEN@GC. a) Fluorescence microscopy images and b) flow cytometry analysis of EMT-6 cells cultured with different concentrations of RhB-labeled HPP/TPEN@GC for 4 h. Scales: 50 μm. c) EMT-6 cell viabilities after being treated with HPP@C, HPP/TPEN@C, HPP@GC and HPP/TPEN@GC at different concentrations in the presence of glucose for 24 h. d) EMT-6 cell viabilities after being treated with HPP@GC and HPP/TPEN@GC at different concentrations in the presence or absence of glucose for 24 h. e) The live (green)/dead (red) dual staining images of EMT-6 cells incubated with HPP@C, HPP/TPEN@C, HPP@GC and HPP/TPEN@GC for 24 h. Scales: 50 μm. f) Quantitative analysis of apoptosis rates and g) flow cytometric results of EMT-6 cells after different treatments. ∗∗p < 0.01, ∗∗∗∗p < 0.0001 versus PBS group; ns, not significant. (For interpretation of the references to colour in this figure legend, the reader is referred to the Web version of this article.)

The cellular uptake of HPP/TPEN@GC was evaluated to confirm that the agents could be delivered into EMT-6 cells. As shown in the fluorescence microscopy images (Fig. 4a and Fig. S17a), red fluorescence signal gradually increased with the increasing concentrations and time of incubation, indicating that cellular uptake efficiency of HPP/TPEN@GC was concentration- and time-dependent. In addition, similar results were also confirmed by flow cytometry (Fig. 4b and Fig. S17b).

The cytotoxicity of HPP/TPEN@GC was assessed by CCK-8 assay on EMT-6 cells. As shown in Fig. 4c, there was nearly no cell damage in the PBS and HPP@C groups, suggesting the good biosafety of HPP@C. Excitingly, at the same concentration, the group treated with HPP/TPEN@GC exhibited the highest cytotoxicity compared to other groups, indicating the optimal synergistic efficacy. Only 19.55 % EMT-6 cells were alive after co-cultured with 10 μg/mL HPP/TPEN@GC for 24 h, while the cell viability rates in the groups treated with the same concentration of HPP/TPEN@C and HPP@GC were 49.35 % and 34.34 %, respectively. The IC_50_ values of HPP/TPEN@GC was calculated to be 4.808 μg/mL. Moreover, the cell viability rates in HPP@GC and HPP/TPEN@GC groups were all obviously decreased in the presence of glucose (Fig. 4d). The observed results can be attributed to the process wherein GOx consumes glucose and catalyzes the generation of surplus H_2_O_2_ through glucose oxidation, and subsequently, the TPEN-Cu(I) copper complex converts the produced H_2_O_2_ into highly toxic ·OH species, which effectively eliminates tumor cells [53,54].

To observe the synergistic cytotoxicity of HPP/TPEN@GC on EMT-6 cells more directly, the live/dead cell staining assay was conducted, in which green fluorescence represented live cells and red fluorescence represented dead cells. As displayed in Fig. 4e, the significant red fluorescence was observed in the group treated with HPP/TPEN@GC. Consistent with the results of CCK-8 assay, cytotoxicity of HPP/TPEN@GC was in a concentration-dependent manner (Fig. S18). Additionally, the results of apoptosis assay also revealed the most severe cell killing ability of HPP/TPEN@GC, in which the apoptosis rate of HPP/TPEN@GC-treated cells was about 85 % (Fig. 4f and g). These results indicated that HPP/TPEN@GC could be successfully delivered into cancer cells and exerted excellent synergistic cytotoxicity.

### 3.6 Metabolic inhibition and ROS generation to induce cell death

**Fig. 5:**
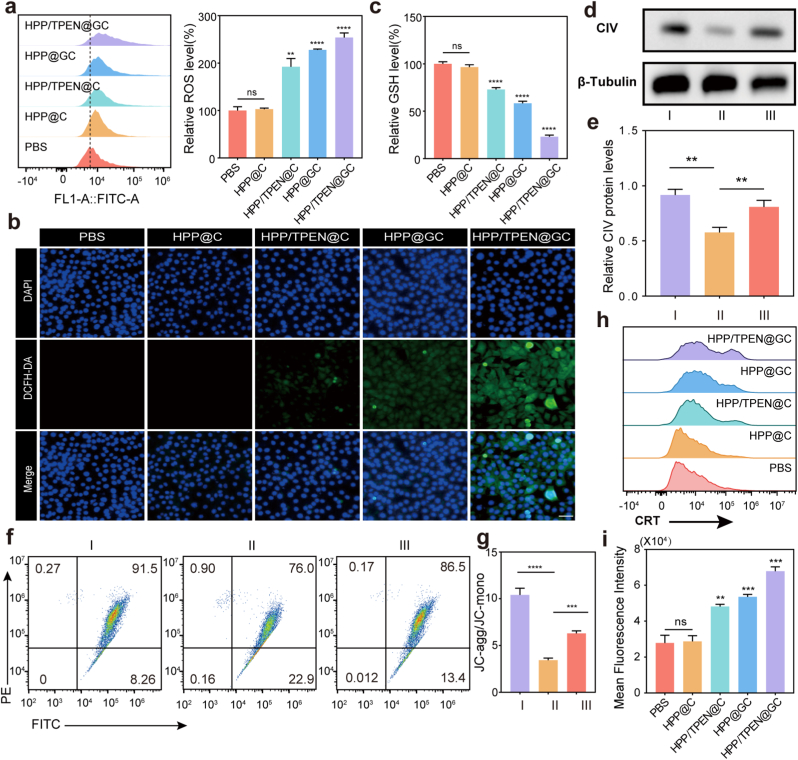
Metabolic inhibition and ROS generation to induce cell death. a) Flow cytometry analysis and corresponding quantitative data of ROS generation after treatment with PBS, HPP@C, HPP/TPEN@C, HPP@GC and HPP/TPEN@GC. b) Fluorescence microscopy images of ROS generation after different treatments for 4 h. Scales: 50 μm. c) Intracellular GSH detection for EMT-6 cells treated with HPP@C, HPP/TPEN@C, HPP@GC and HPP/TPEN@GC for 4 h. d) Western blot results and e) corresponding statistical data of CIV after different treatments: I, PBS; II, HPP/TPEN@GC; III, HPP/TPEN@GC+10 μM Cu(II). Complex IV is referred to as CIV. β-Tubulin was used as a control. f) Flow cytometric results of MMP of EMT-6 cells treated as indicated using JC-1 dye and (g) quantitative analysis of JC-agg/JC-mono. I, PBS; II, HPP/TPEN@GC; III, HPP/TPEN@GC+10 μM Cu(II). MMP is evaluated based on ratio of JC-1 aggregates (JC-agg, red fluorescence)/JC-1 monomers (JC-mono, green fluorescence). (h) Flow cytometric assay and (i) the corresponding quantitative analysis of CRT expression. ∗∗p < 0.01, ∗∗∗p < 0.001, ∗∗∗∗p < 0.0001 versus PBS group; ns, not significant. (For interpretation of the references to colour in this figure legend, the reader is referred to the Web version of this article.)

To evaluate the effect of HPP/TPEN@GC on the glucose consumption and the cellular redox state, the intracellular ROS generation was detected. Quantitative analysis of fluorescence intensity by flow cytometry revealed a 2.5-fold increase in HPP/TPEN@GC-treated cells compared to controls (Fig. 5a). And the fluorescence intensity in HPP/TPEN@C- and HPP@GC-treated cells were 1.9-fold and 2.3-fold higher than that in the control, respectively. The increases in the groups of HPP@GC- and HPP/TPEN@GC-treated cells were probably attributed to the accumulation of H_2_O_2_ catalyzed by GOx, followed by the subsequent generation of highly reactive ·OH catalyzed by TPEN-Cu(I). The catalytic production of H_2_O_2_ by GOx relies on the presence of glucose as a reactant. Therefore, the accumulation of H_2_O_2_ was often accompanied by glucose starvation, which could also contribute to cell death (Fig. 4c) [55,56]. Besides, the strongest green fluorescence signals were recorded by fluorescence microscopy in the HPP/TPEN@GC group, while nearly no signals could be detected in the PBS and HPP@C groups (Fig. 5b). As expected, the ability of HPP/TPEN@GC to enhance intracellular ROS generation was concentration-dependent (Fig. S19). Furthermore, after 4 h of treatments, the concentration of GSH in HPP/TPEN@GC-treated cells decreased by 77 % compared with the PBS group and HPP/TPEN@GC showed the strongest GSH consuming capacity (Fig. 5c). There are two primary factors contributing to this phenomenon. Firstly, GOx-induced glucose consumption and TPEN-induced copper depletion disrupt the energy supply of tumor cells and partially inhibit the synthesis of GSH [57]. Secondly, TPEN, functioning as a copper chelator, chelates copper ions to form TPEN-Cu(II), which is subsequently reduced to TPEN-Cu(I) while consuming GSH [40]. As demonstrated by the aforementioned results, HPP/TPEN@GC exhibited a synergistic role between GOx and TPEN in ·OH production and GSH depletion for enhanced CDT.

Next, to further explore the mechanism of TPEN, the expression of CIV was quantified by western blot analysis. CIV is an essential enzyme of the mitochondrial respiratory chain, for which copper ions serve as an important cofactor [21]. As expected, an obvious reduction of CIV protein levels was observed in HPP/TPEN@GC-treated EMT-6 cells compared to controls (Fig. 5d and e). The expression of CIV was restored following the supplementation of copper. The similar results could also be observed in TPEN-treated cells, which suggested that TPEN could interfere with the assembly and function of CIV (Fig. S20). Besides, the mitochondrial function was evaluated using JC-1 dye. Compared with the PBS group, the cells incubated with HPP/TPEN@GC produced stronger green fluorescence (JC-1 monomers), indicating the mitochondrial membrane potential (MMP) was decreased (Fig. 5f and g). Similar to the results of western blot analysis, the ratio of JC-1 aggregates/JC-1 monomers was significantly increased when extra copper was supplied (Fig. 5f and g). These results demonstrated the influence of HPP/TPEN@GC on the mitochondrial function due to copper depletion. According to the flow cytometry analysis of MMP assay, the cells treated with HPP/TPEN@C exhibited weaker red fluorescence than the cells treated with HPP@GC, suggesting that TPEN brought greater damage to mitochondria than GOx (Fig. S21). Taken together, the mechanism underlying HPP/TPEN@GC-induced cell death involved the disruption of glucose metabolism, as well as the disturbance of redox balance in cancer cells.

Additionally, CDT was supposed to trigger immunogenic cell death (ICD) of the tumor cells [58]. During the process of ICD, calreticulin (CRT) translocates to the cell surface, where it functions as an “eat-me” signal to recruit and activate antigen-presenting cells, thereby promoting immune response activation [59]. In the presence of HPP/TPEN@C or HPP@GC, CRT expression on the surface of tumor cells was significantly higher than that in control group and HPP@C group (Fig. 5h and i). The highest level of CRT expression was observed in the HPP/TPEN@GC group. The trend was consistent with ROS production, suggesting that HPP/TPEN@GC could activate immunity by promoting ROS generation.

### 3.7 In vivo biodistribution of HPP/TPEN@GC

**Fig. 6:**
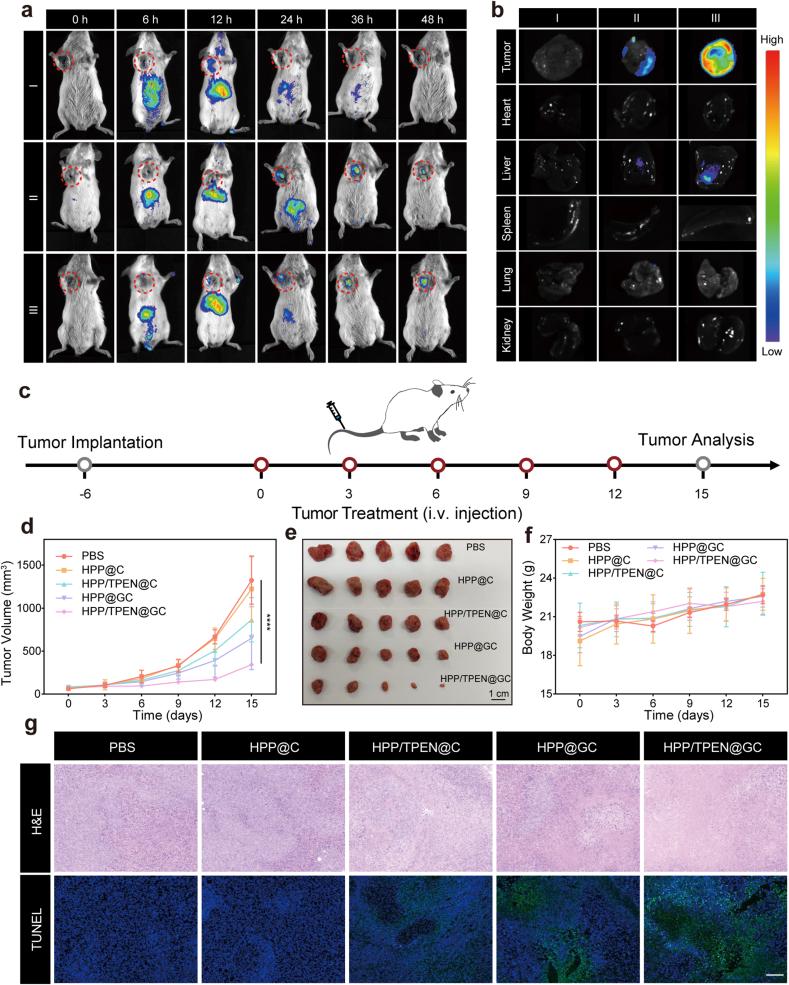
*In vivo* biodistribution and antitumor activity of HPP/TPEN@GC. a) Fluorescence images of tumor bearing mice post intravenous injection of ICG (I), ICG-HPP/TPEN@G (II) and ICG-HPP/TPEN@GC (III), respectively. b) Fluorescence images of dissected tumors and major organs after intravenous injection of ICG (I), ICG-HPP/TPEN@G (II) and ICG-HPP/TPEN@GC (III) for 48 h. c) Schematic illustration of antitumor experiment using EMT-6 tumor bearing mice model. d) Tumor volume curves of mice after different administrations. e) The representative photos of tumors from mice in different groups. f) The body weight curves of mice after different administrations. g) H&E and TUNEL staining images of tumors in different groups. Scales: 50 μm. ∗∗p < 0.01 versus PBS group.

To verify the tumor-targeting capacity and accumulation behavior of HPP/TPEN@GC, an in vivo biodistribution study was conducted using a fluorescence imaging system. As presented in Fig. 6a, after intravenous injection, free ICG (I) rapidly appeared in the tumor site at 12 h and then rapidly disappeared. By contrast, after ICG-HPP/TPEN@GC (III) injection, the fluorescence signal was detected in tumor tissues at 12 h and the intensity gradually increased over time (Fig. 6a). Even 48 h post injection, the strongest fluorescence signal was still observed in isolate tumors of ICG-HPP/TPEN@GC group (Fig. 6b). Comparatively, for ICG-HPP/TPEN@G (II), the fluorescence intensity in tumor site reached the maximum at 36 h and then gradually eliminated. All the above data indicated that HPP/TPEN@GC possessed a desirable capacity for tumor targeting and exhibited an extended duration of retention within tumor tissues.

### 3.8 In vivo antitumor activity of HPP/TPEN@GC

Based on the results of in vivo biodistribution experiments, the antitumor experiment was performed as shown in Fig. 6c. After five cycles of treatments, the average tumor volume in the PBS group was almost four times that of the HPP/TPEN@GC group (Fig. 6d). According to the tumor volume curves, HPP/TPEN@GC exhibited the most remarkable tumor suppression effect. Consistent with the results of the tumor volume curves, weight of tumors from mice in different groups followed similar patterns (Fig. 6e and Fig. S22). Meanwhile, there were no significant differences in the body weight of mice across different groups (Fig. 6f). Furthermore, H&E staining and TUNEL staining were performed on tumor tissues. In H&E staining images, the largest necrotic area was observed in HPP/TPEN@GC group (Fig. 6g). Consistently, TUNEL images also exhibited the most severe damage in HPP/TPEN@GC-treated tumors (Fig. 6g). Collectively, HPP/TPEN@GC exhibited exceptional effectiveness in suppressing tumor growth.

### 3.9 In vivo antitumor immune response

**Fig. 7:**
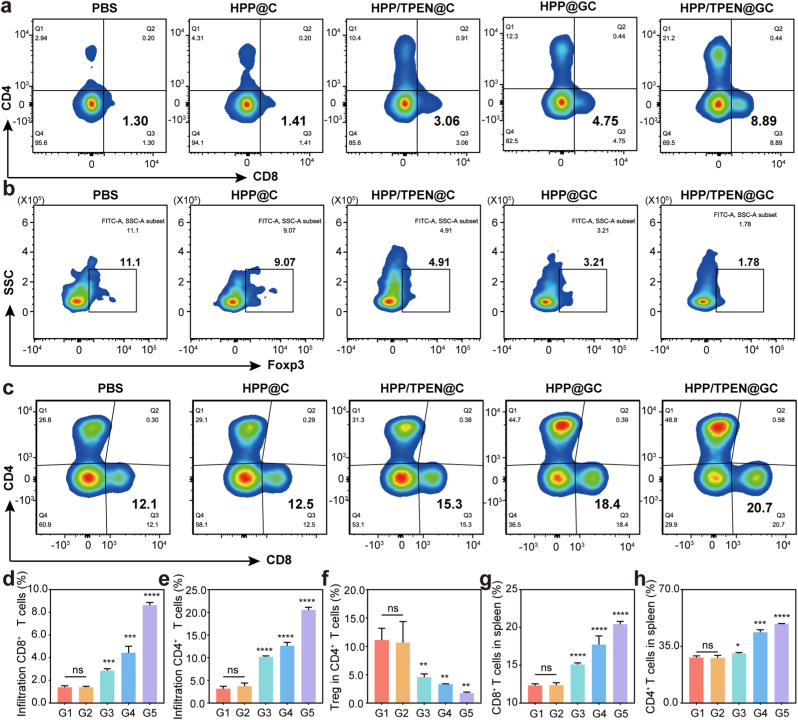
*In vivo* antitumor immune response. a) Representative flow cytometry plots of CD4^+^ and CD8^+^ T cells (gated on CD3^+^ T cells) in the tumor tissues after different treatments. b) Representative flow cytometry plots Tregs (gated on CD4^+^ T cells) in the tumor tissues after different treatments. c) Representative flow cytometry plots of CD4^+^ and CD8^+^ T cells (gated on CD3^+^ T cells) in the spleen tissues after different treatments. (d–f) The quantitative analysis of d) CD8^+^ T cells, e) CD4^+^ T cells and f) Tregs in tumor tissues. (g–h) The quantitative analysis of g) CD8^+^ T cells and h) CD4^+^ T cells in spleen tissues. G1: PBS; G2: HPP@C; G3: HPP/TPEN@C; G4: HPP@GC; G5: HPP/TPEN@GC. ∗p < 0.05, ∗∗p < 0.01, ∗∗∗p < 0.001, ∗∗∗∗p < 0.0001 versus PBS group; ns, not significant.

After confirming the promising tumor therapeutic effect, the in vivo immune response induced by HPP/TPEN@GC was further investigated. Tumor and spleen tissues were dissociated into single-cell suspensions, and immune cells were subsequently analyzed by flow cytometric analysis. After the treatment of HPP/TPEN@C and HPP@GC, the amount of CD4^+^ and CD8^+^ T cells at the tumor site increased to different extents (Fig. 7a). Notably, the amounts of CD4^+^ and CD8^+^ T cells in the HPP/TPEN@GC group were ∼4.9 times and ∼6.3 times higher, respectively, than those in the control group and HPP@C group, which suggested that HPP/TPEN@GC could activate immune cells, thereby more effectively inhibiting tumor development (Fig. 7d and e). Meanwhile, regulatory T cells (Treg cells) in the tumor tissues were obviously restricted, indicating that HPP/TPEN@GC could modulate the immunosuppressive tumor microenvironment to enhance the therapeutic effect (Fig. 7b and f). Compared to the control group and HPP@C group, the populations of CD4^+^ and CD8^+^ T cells in the spleens were also notably increased, confirming the activation of systemic immunity (Fig. 7c, g and h). All the experimental results validated that HPP/TPEN@GC could activate the immune system by inducing the production of ROS, thereby effectively inhibiting tumor growth and development.

### 3.10 In vivo biosafety assessment of HPP/TPEN@GC

**Fig. 8:**
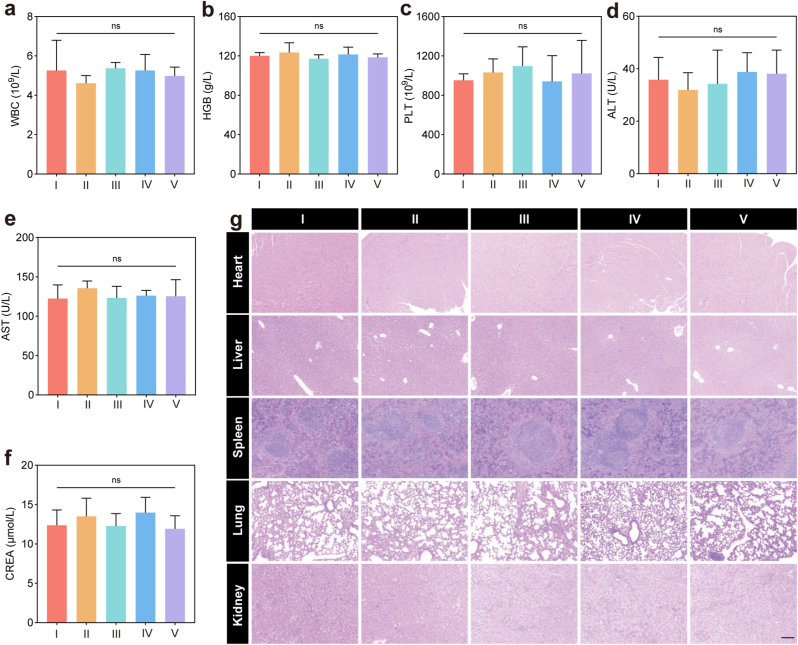
*In vivo* biosafety assessment of HPP/TPEN@GC. a) White blood cell count (WBC), b) hemoglobin (HGB), c) platelet (PLT), d) alanine aminotransferase (ALT), e) aspartate aminotransferase (AST) and f) creatinine (CREA) levels recorded for blood samples of mice in different treatment groups: I, PBS; II, HPP@C; III, HPP/TPEN@C; IV, HPP@GC; V, HPP/TPEN@GC. g) H&E staining images of major organs (heart, liver, spleen, lung and kidney) in different groups: I, PBS; II, HPP@C; III, HPP/TPEN@C; IV, HPP@GC; V, HPP/TPEN@GC. Scales: 50 μm ns, not significant.

It was necessary to evaluate biosecurity of HPP/TPEN@GC for its potential future applications. No outliers were identified in blood routine testing, including white blood cell count (WBC), hemoglobin (HGB) and platelet (PLT) (Fig. 8a–c). And, no abnormalities were found on serum analysis including alanine aminotransferase (ALT), aspartate aminotransferase (AST) and creatinine (CREA), indicating the administration of HPP/TPEN@GC did not result in any noticeable impairment of hepatic or renal functions. (Fig. 8d–f). Additionally, no detectable pathological injury was found in the major organs from HPP/TPEN@GC-treated mice by H&E staining (Fig. 8g). Based on the aforementioned results, it was evident that HPP/TPEN@GC exhibited exceptional biocompatibility and was devoid of any adverse side effects. These findings strongly support its potential for safe and effective clinical applications.

## 4 Conclusions

In summary, we have successfully developed a TPEN and GOx co-loaded cascade nanoreactor (HPP/TPEN@GC) to reduce the availability of endogenous copper and glucose for cancer starvation and chemodynamic therapy. After intravenous injection, the nanoreactor targeted tumor cells by virtue of the interaction between CS and overexpressed CD44 receptor. Subsequently, the released TPEN and GOx reduced the availability of endogenous copper and glucose, resulting in the blockade of mitochondrial OXPHOS and glycolysis, thus depriving the tumor cells of the necessary energy for rapid proliferation. Inhibiting both OXPHOS and glycolysis could avoid therapeutic inefficacy caused by metabolic switching between these two pathways. Concurrently, the generated TPEN-Cu(I) and H_2_O_2_ became potential anti-tumor agents and could further react to produce ·OH for enhanced CDT. Collectively, HPP/TPEN@GC achieved highly effective therapeutic effect with excellent biosafety and biocompatibility. The novel strategy of reducing the availability of endogenous copper offers a new perspective and holds great potential in cancer therapy.

## CRediT authorship contribution statement

**Chunhui Wang:** Writing – review & editing, Writing – original draft, Visualization, Validation, Methodology, Investigation, Formal analysis, Conceptualization. **Pingting Ye:** Writing – original draft, Validation, Methodology, Investigation, Formal analysis. **Mengyao Chen:** Visualization, Investigation, Formal analysis. **Ruihao Li:** Investigation, Formal analysis. **Yixuan Wen:** Investigation, Formal analysis. **Yu Wang:** Investigation, Formal analysis. **Xiaohan Tong:** Investigation, Formal analysis. **Chunyan Dong:** Supervision, Resources, Funding acquisition. **Shuo Shi:** Writing – review & editing, Supervision, Resources, Funding acquisition, Conceptualization.

## Ethics approval and consent to participate

Protocols of animal experiments included in this study were approved by the Institutional Animal Care and Use Committee of Tongji University.

## Consent for publication

All authors consent to publish.

## Declaration of competing interest

The authors declare that they have no known competing financial interests or personal relationships that could have appeared to influence the work reported in this paper.

## Data Availability

Data will be made available on request.
