# Supplementary material for: Reducing the availability of endogenous copper and glucose for cascade starvation therapy and chemodynamic therapy
Source: Mater Today Bio. 2025 Mar 24;32:101702. doi: 10.1016/j.mtbio.2025.101702 (PMC11995123; doi:10.1016/j.mtbio.2025.101702)
Supplement: Multimedia component 1 [file mmc1.docx]

Supporting Information

**Reducing** **the** **availability of endogenous copper and glucose for** **cascade starvation therapy and** **chemodynamic therapy**

*Chunhui Wang†, Pingting Ye†, Mengyao Chen, Ruihao Li, Yixuan Wen, Yu Wang, Xiaohan Tong, Chunyan Dong** *and Shuo Shi**

***Corresponding author. E-mail: shishuo@tongji.edu.cn, cy_dong@tongji.edu.cn.

†These authors contributed equally to this work.

**Table S1.** The differential expression analysis of genes associated with copper uptake and transport.

| gene | logFC | pvalue | fdr |
| --- | --- | --- | --- |
| SLC31A1 | 3.284476 | 2.46E-06 | 2.71E-06 |
| COX17 | 7.921531 | 9.12E-33 | 3.34E-32 |
| SCO1 | -0.62054 | 4.96E-14 | 7.79E-14 |
| SCO2 | 0.142969 | 5.34E-17 | 1.47E-16 |
| COA6 | 5.162615 | 1.25E-52 | 1.38E-51 |
| CCS | 6.498539 | 6.58E-13 | 9.05E-13 |
| SOD1 | 35.60516 | 1.61E-15 | 2.96E-15 |
| ATOX1 | 5.360697 | 3.89E-34 | 2.14E-33 |
| ATP7A | -0.63826 | 4.48E-06 | 4.48E-06 |
| ATP7B | 0.901069 | 2.31E-06 | 2.71E-06 |


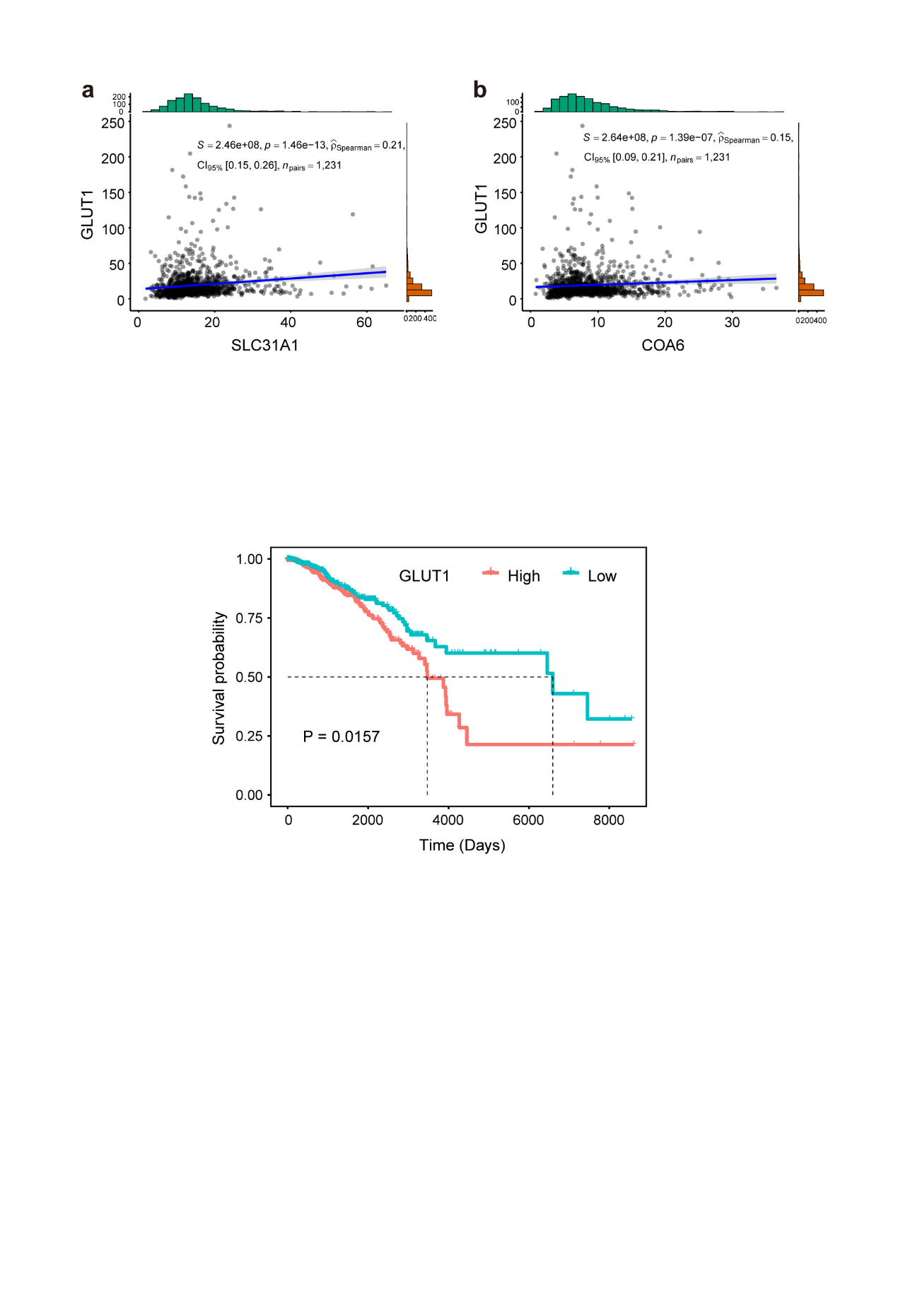


**Figure S1.** (a) Correlation analysis of SLC31A1 and GLUT1; (b) Correlation analysis of COA6 and GLUT1 according to the data from TCGA-BRCA dataset.


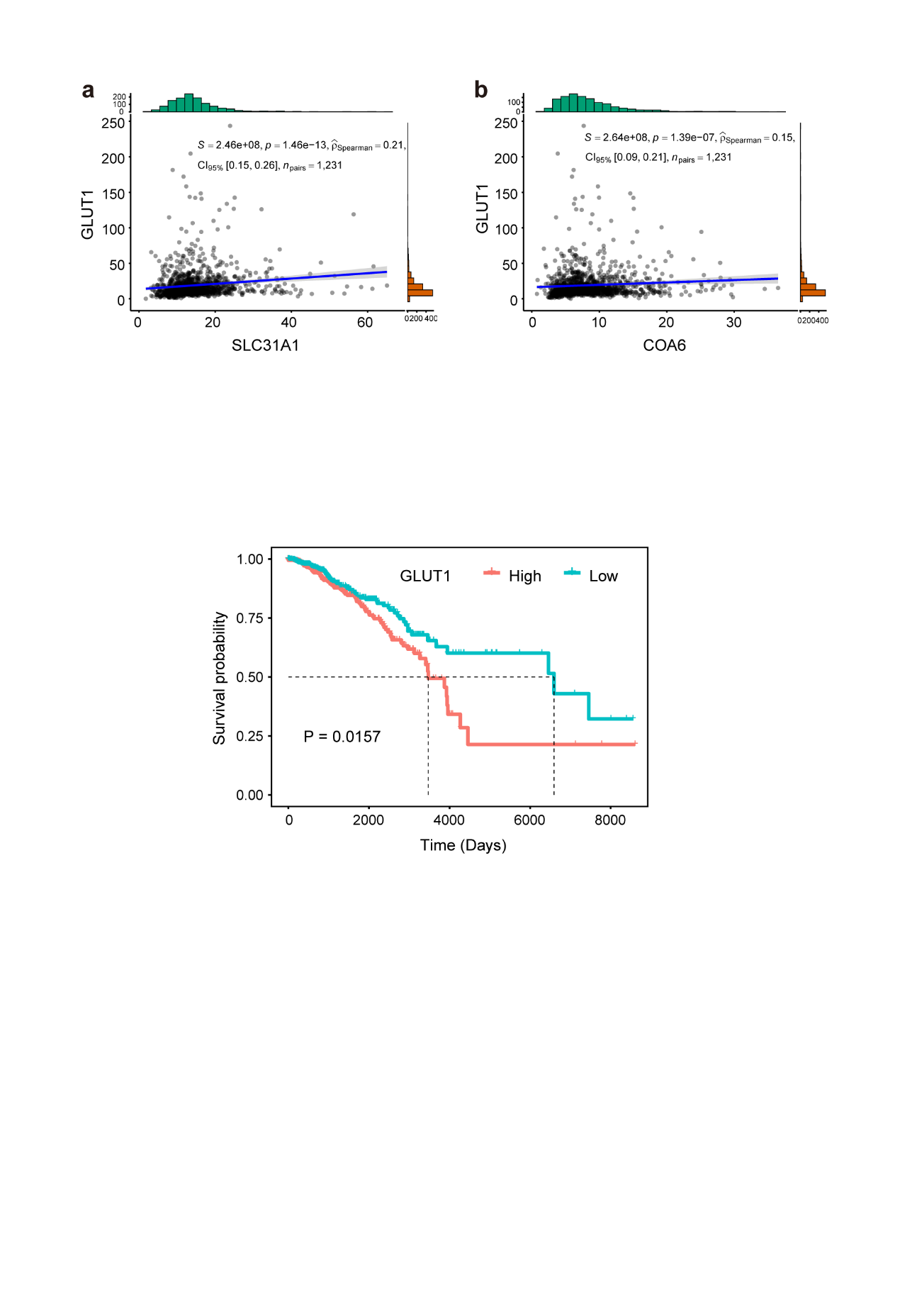
**Figure S2.** Effects of GLUT1 expression on survival time of patients with BRCA.


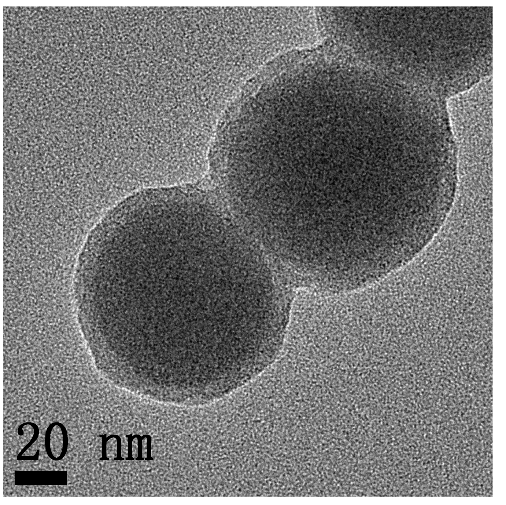
**Figure S3.** The TEM image of SiO_2_@PDA.


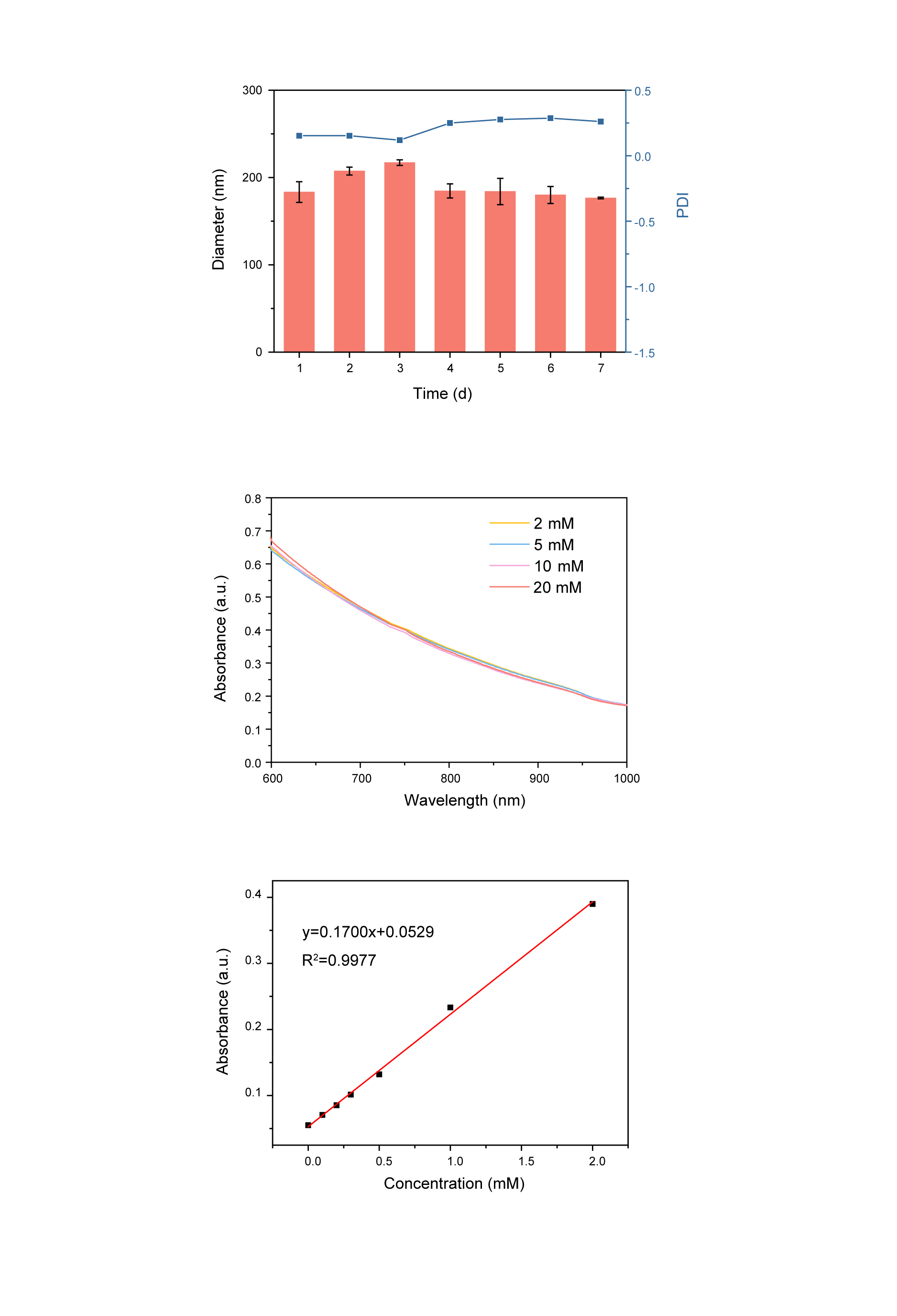


**Figure S4.** Hydrodynamic diameters and PDI of HPDA nanoparticles measured at different time points.


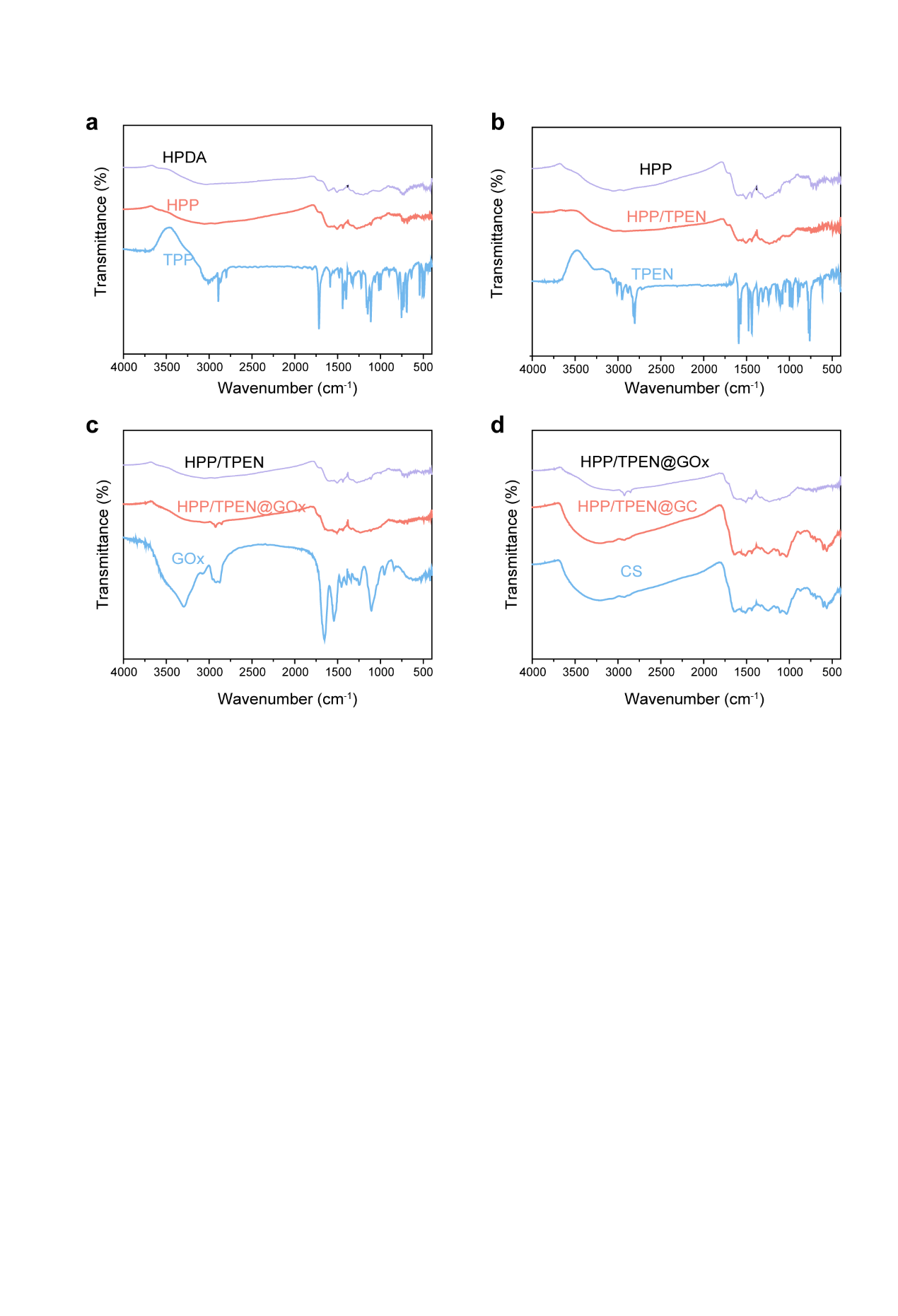


**Figure S5.** (a) The FT-IR spectra of HPDA, HPP and TPP; (b) the FT-IR spectra of HPP, HPP/TPEN and TPEN; (c) the FT-IR spectra of HPP/TPEN, HPP/TPEN@G and GOx, and (d) the FT-IR spectra of HPP/TPEN@G, HPP/TPEN@GC and CS.


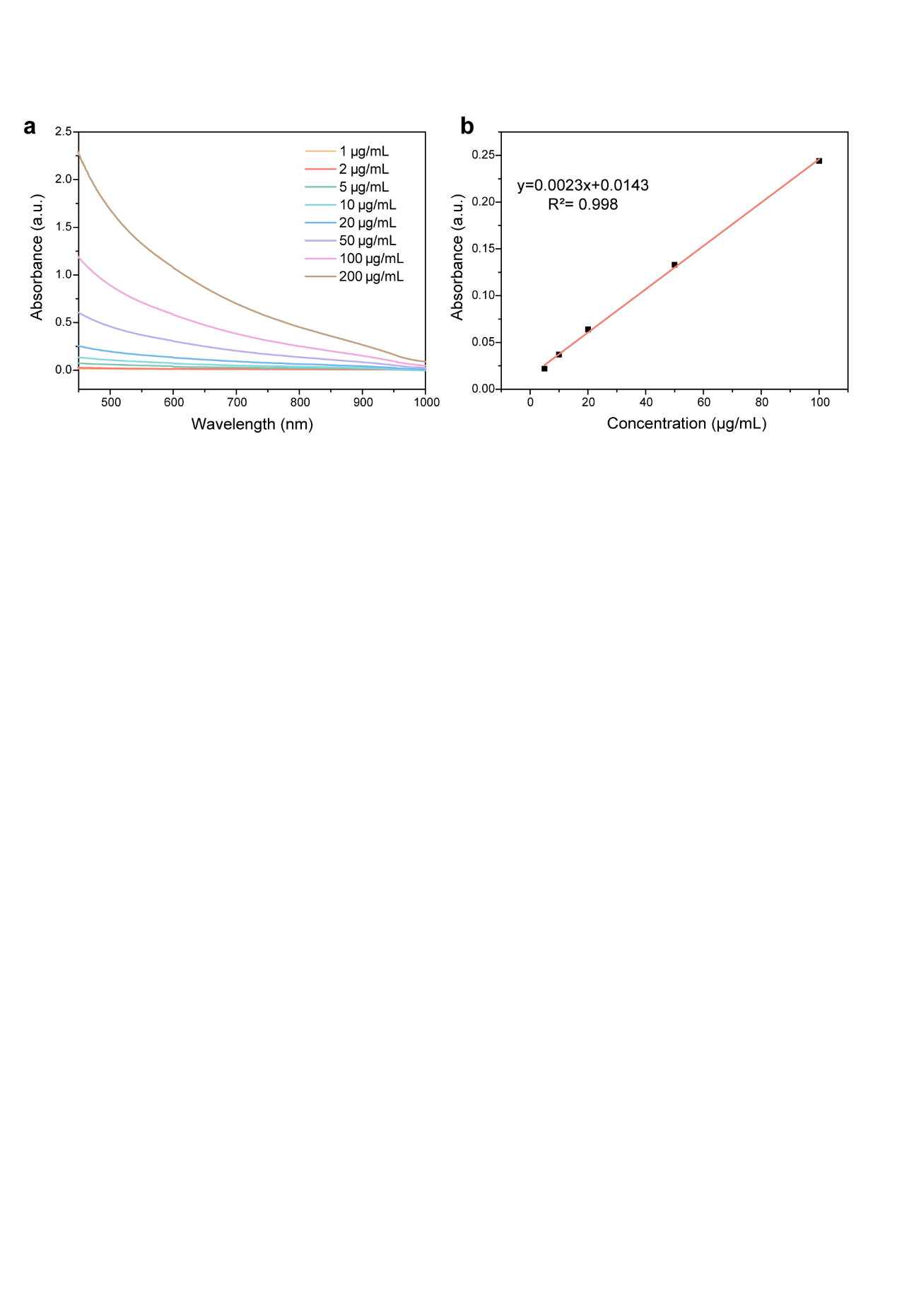


**Figure S6.** The UV-Vis adsorption spectra and the standard curve of HPDA.


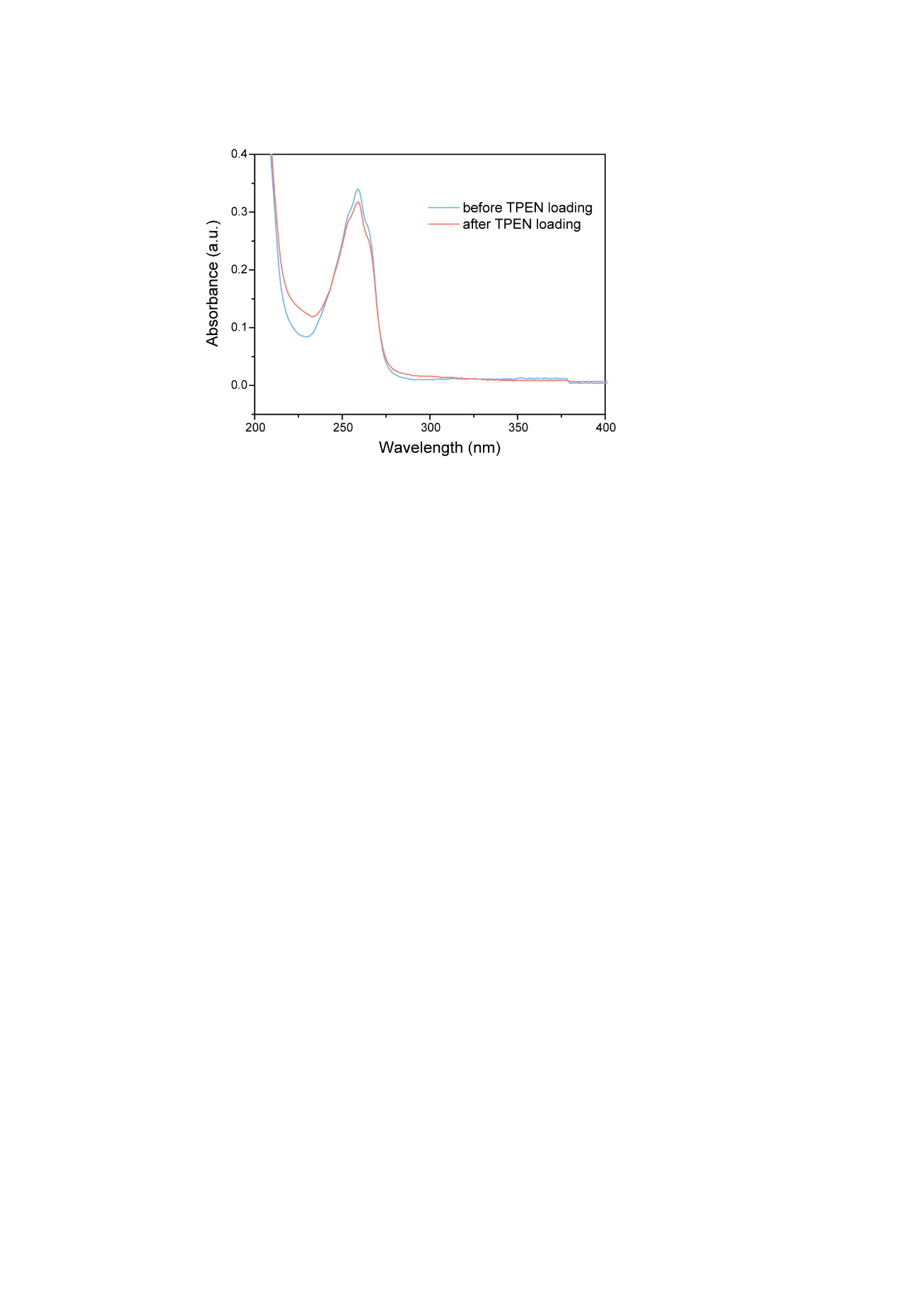


**Figure S7.** The UV-Vis adsorption spectra of the supernatant (1:100 in ethanol) before and after TPEN loading.


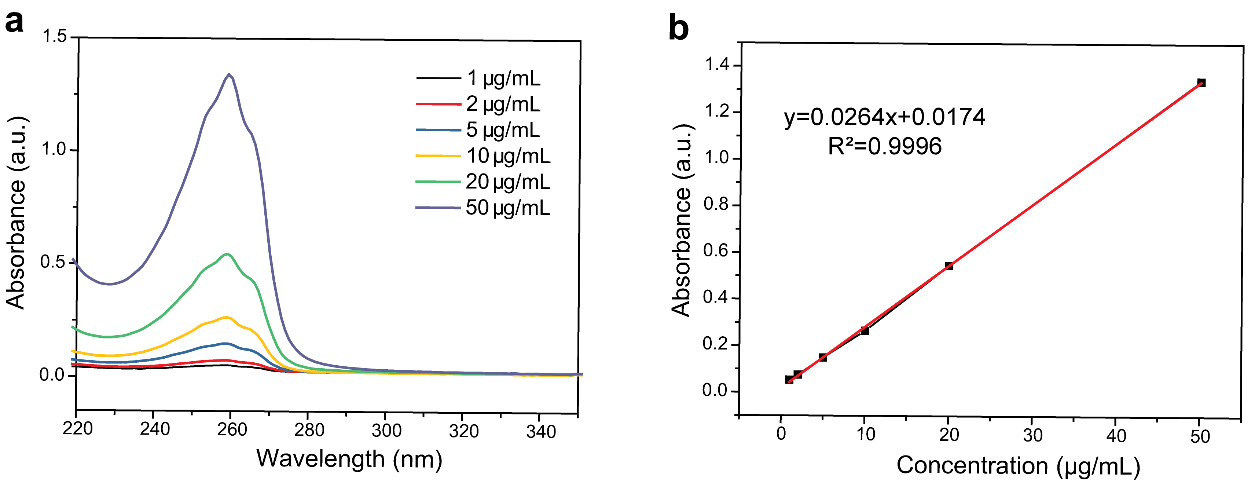


**Figure S8.** The UV-Vis absorption spectra and the standard curve of TPEN.


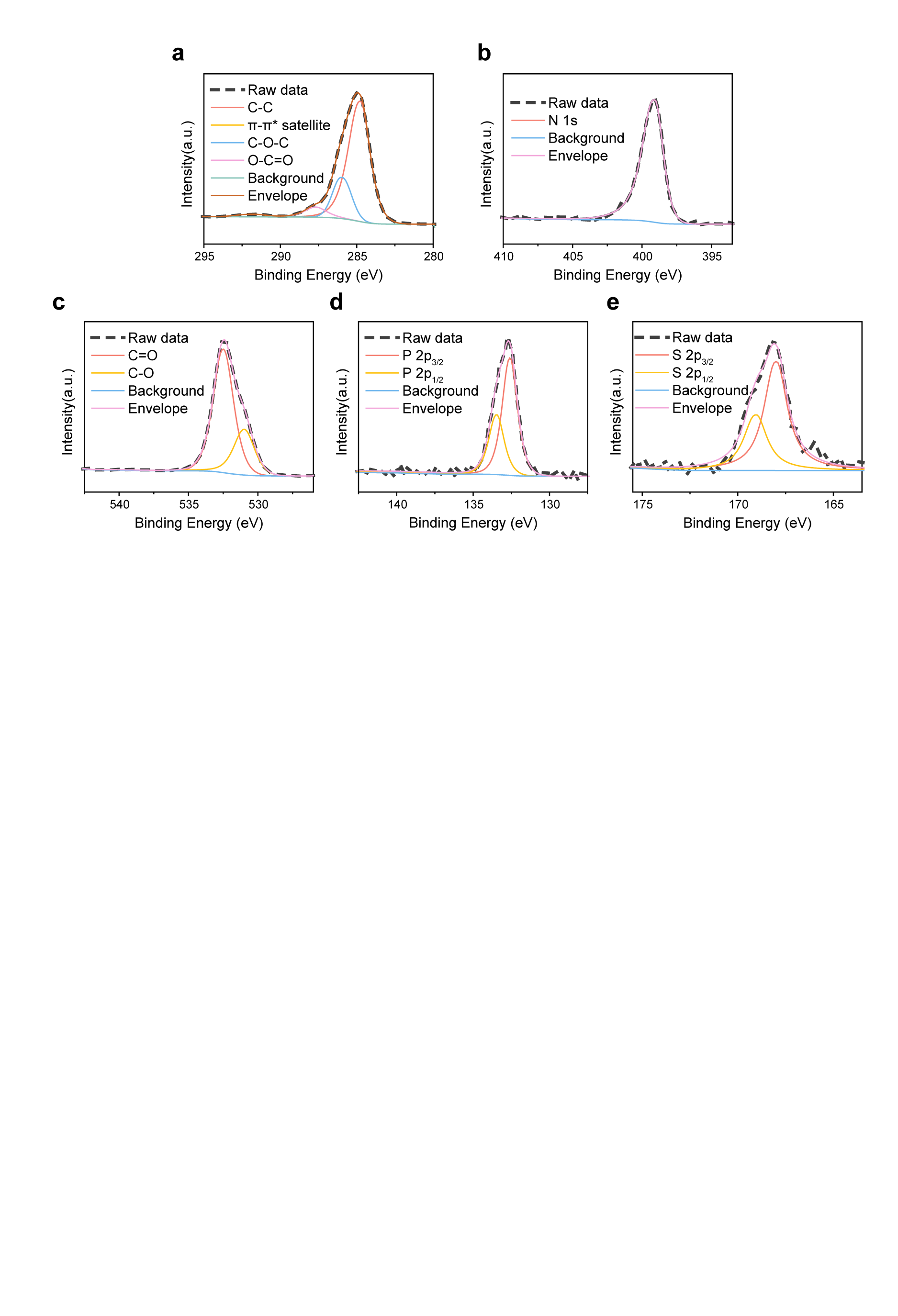


**Figure S9.** The high-resolution XPS spectra of (a) C 1s, (b) N 1s, (c) O 1s, (d) P 2p and (e) S 2p.


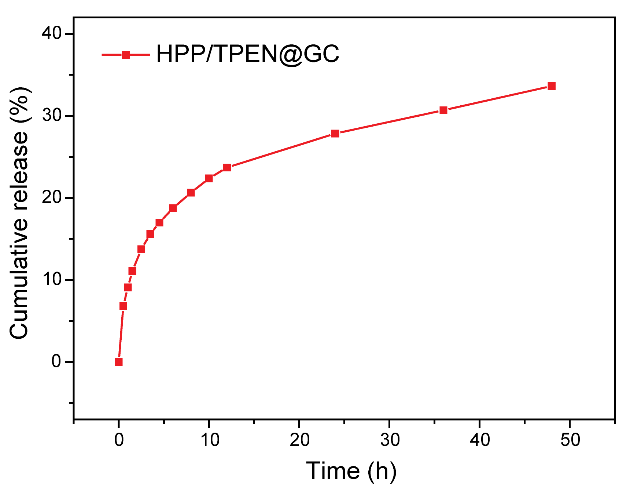


**Figure S10.** The cumulative release of TPEN during 48 h.


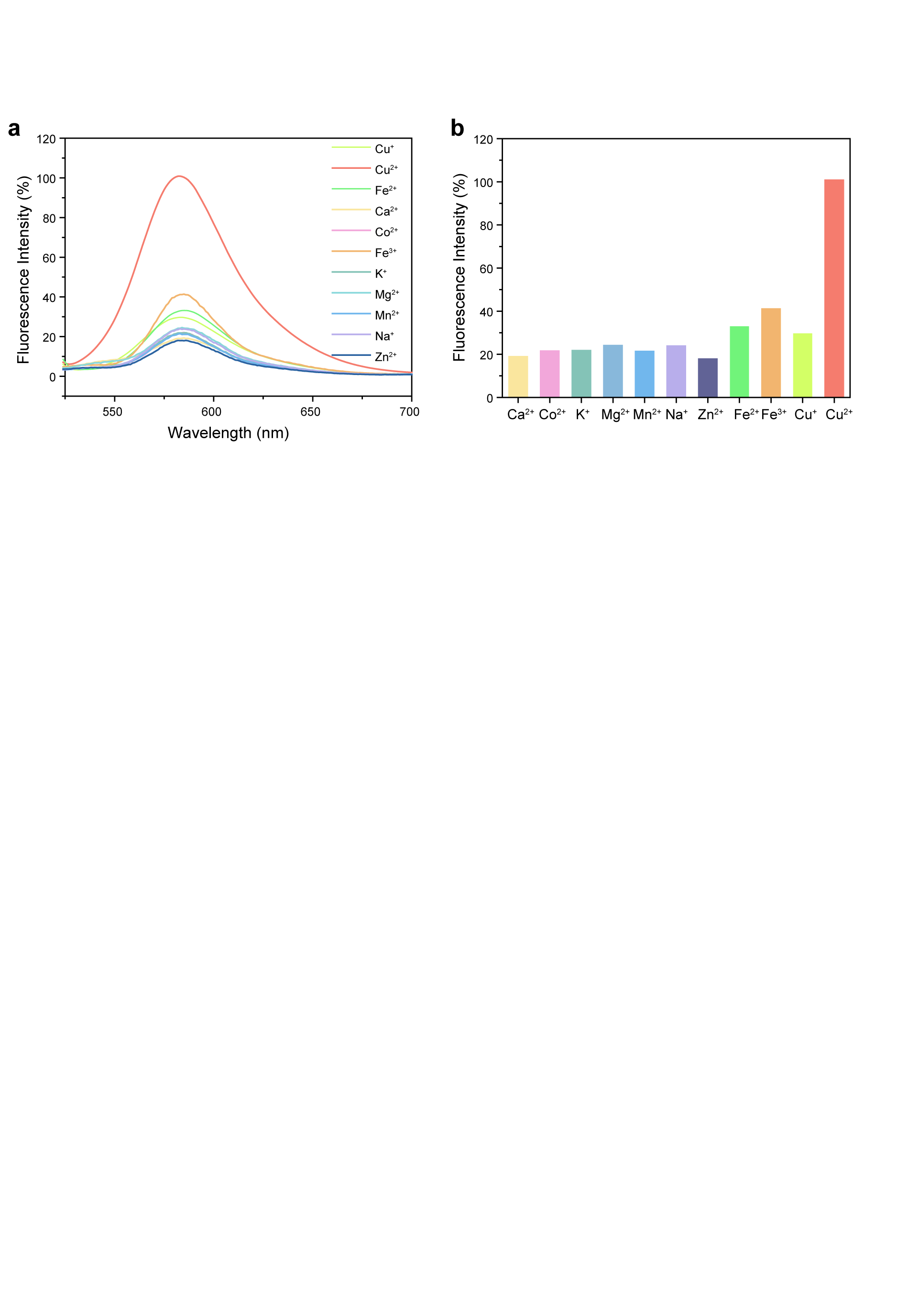


**Figure S11.** (a)The fluorescence spectra of RBH after incubated with different metal ions and (b) the relative intensity of the peak.


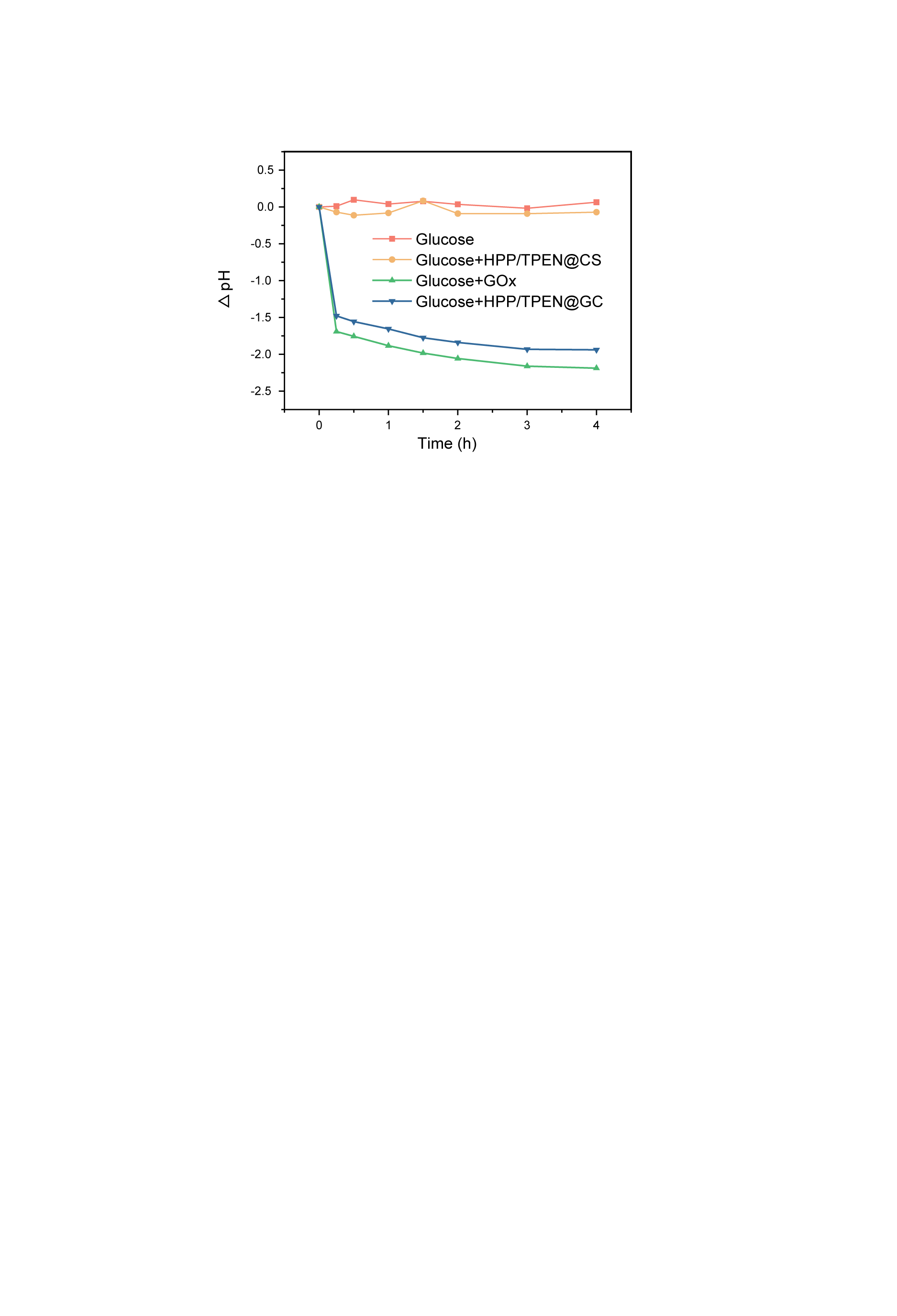


**Figure S12.** The time-dependent pH changes of the glucose solution after different treatments.


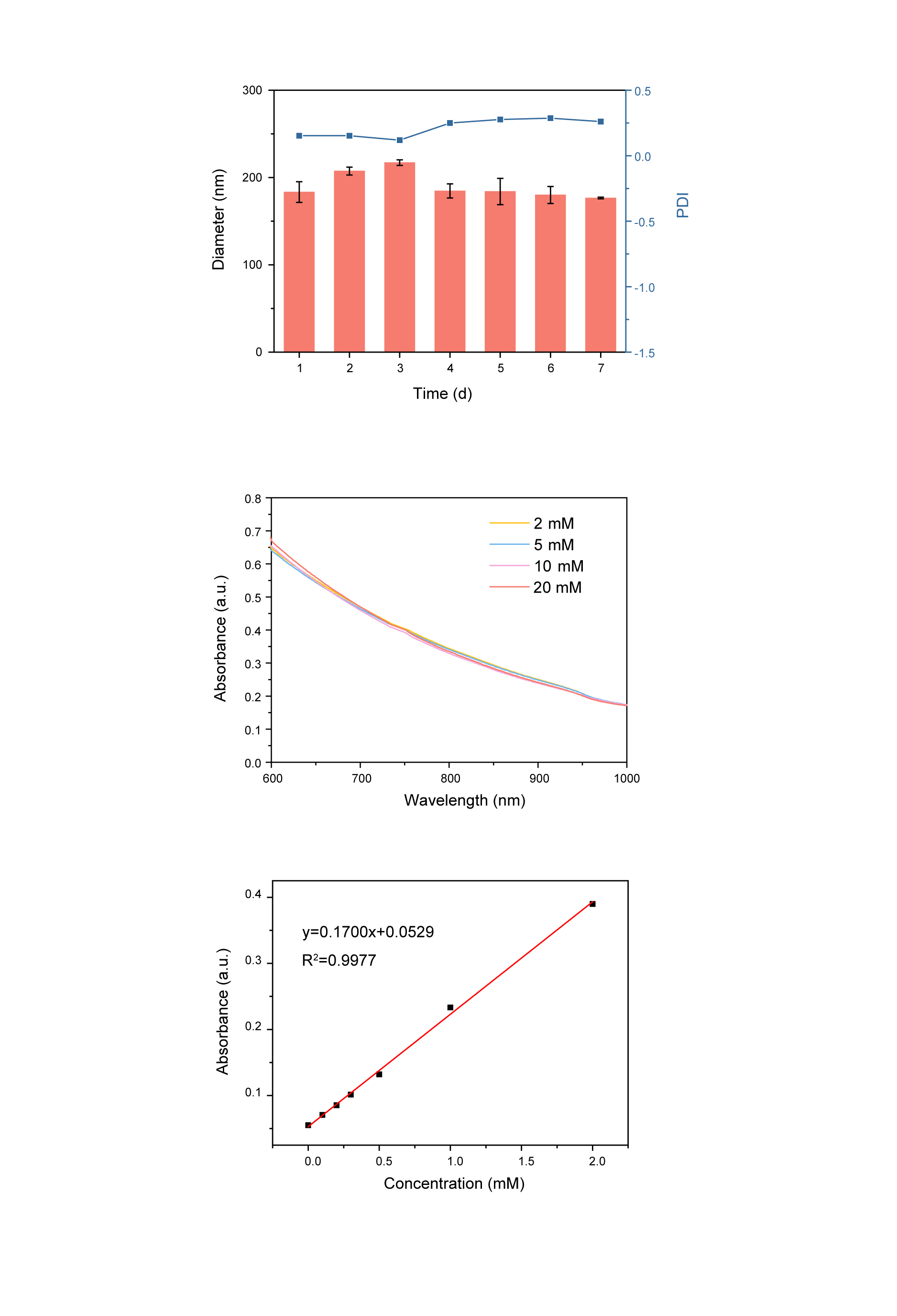


**Figure S13.** The standard curve of H_2_O_2_.


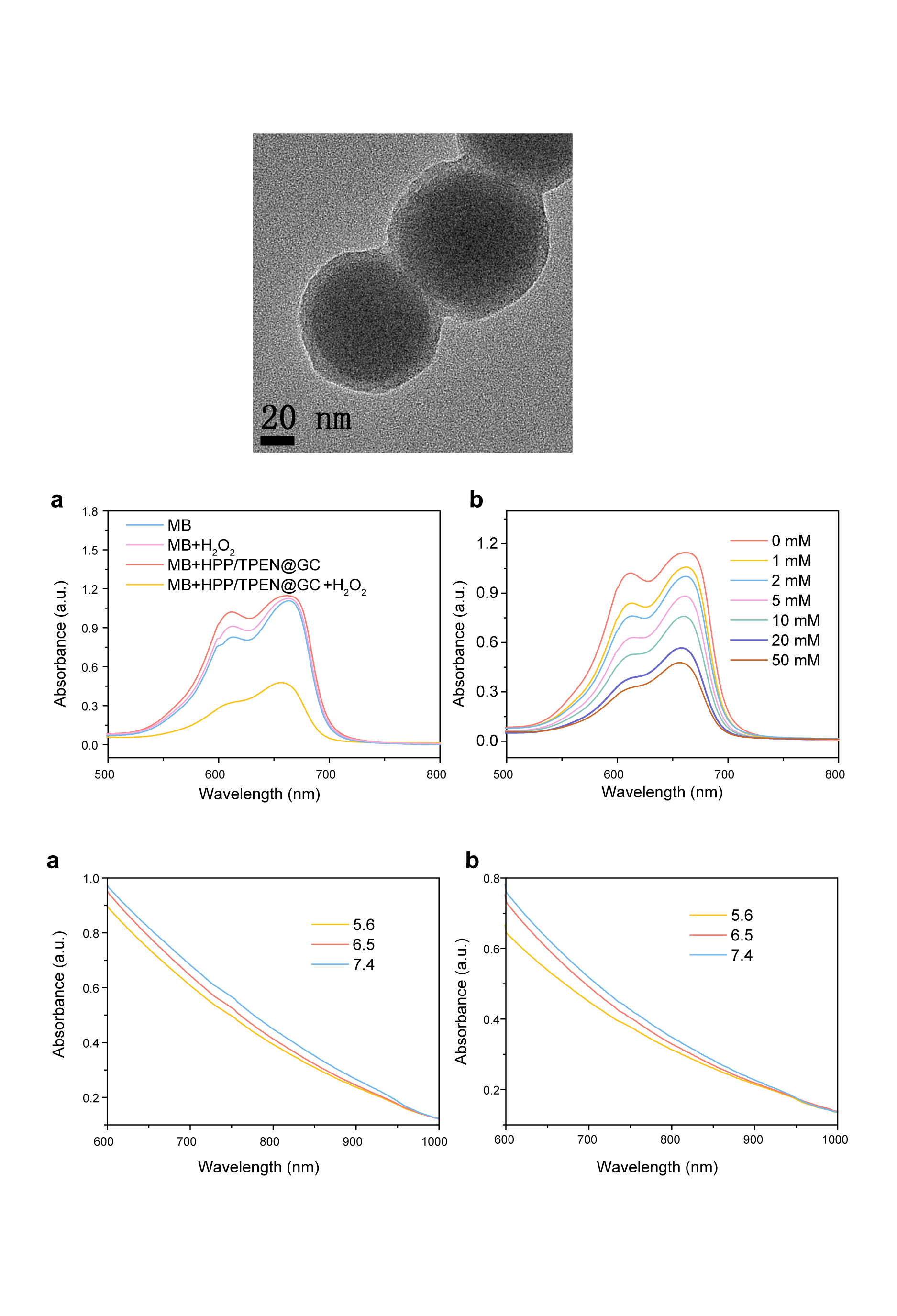


**Figure S14.** (a) UV-Vis spectra of MB after treated with different formulations. (b) UV-Vis spectra of MB after incubated with HPP/TPEN@GC (which was pretreated with copper) and different concentrations of H_2_O_2_.


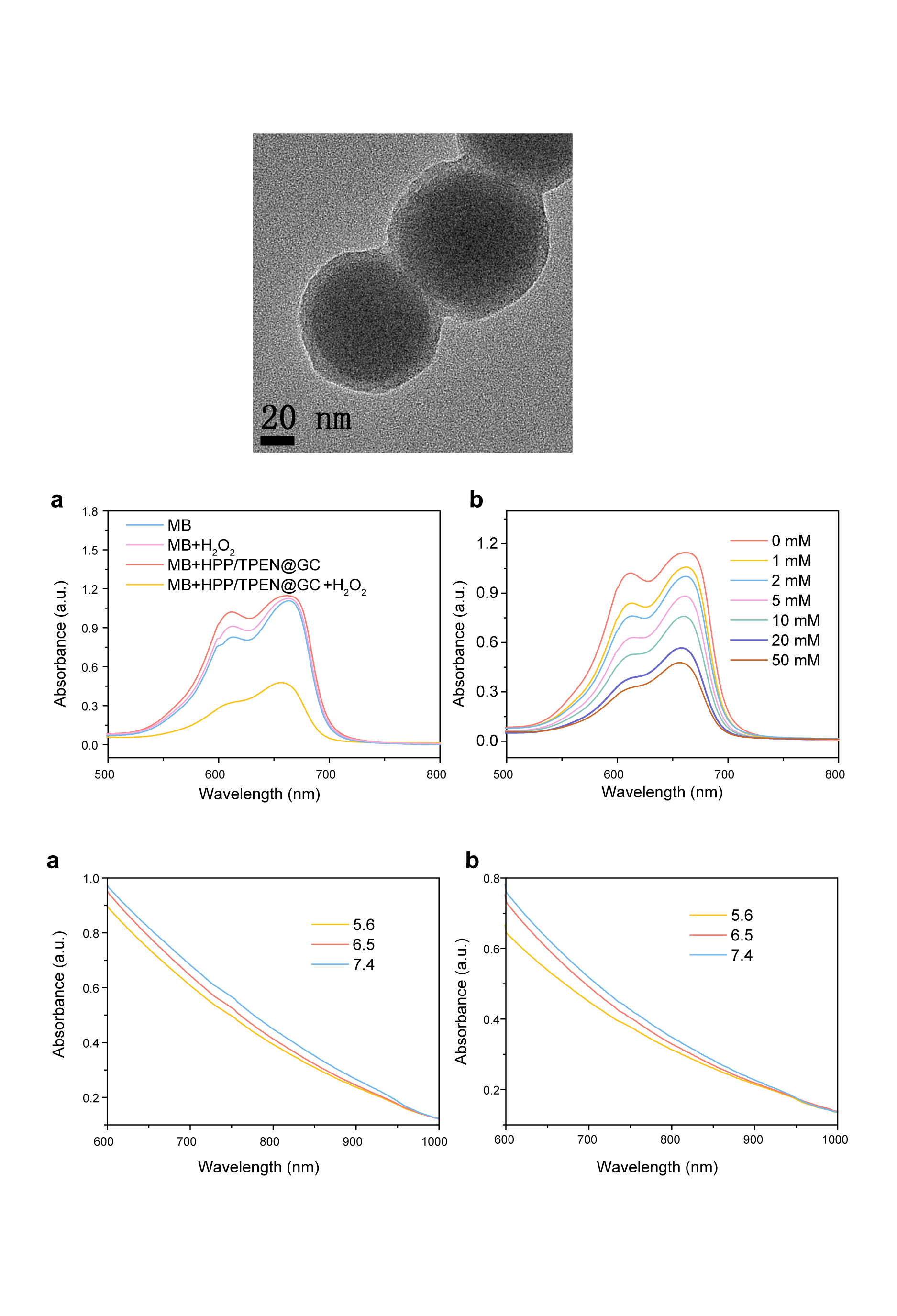


**Figure S15.** UV-Vis spectra of (a) HPDA and (b) HPP/TPEN@CS after incubated at different pH.


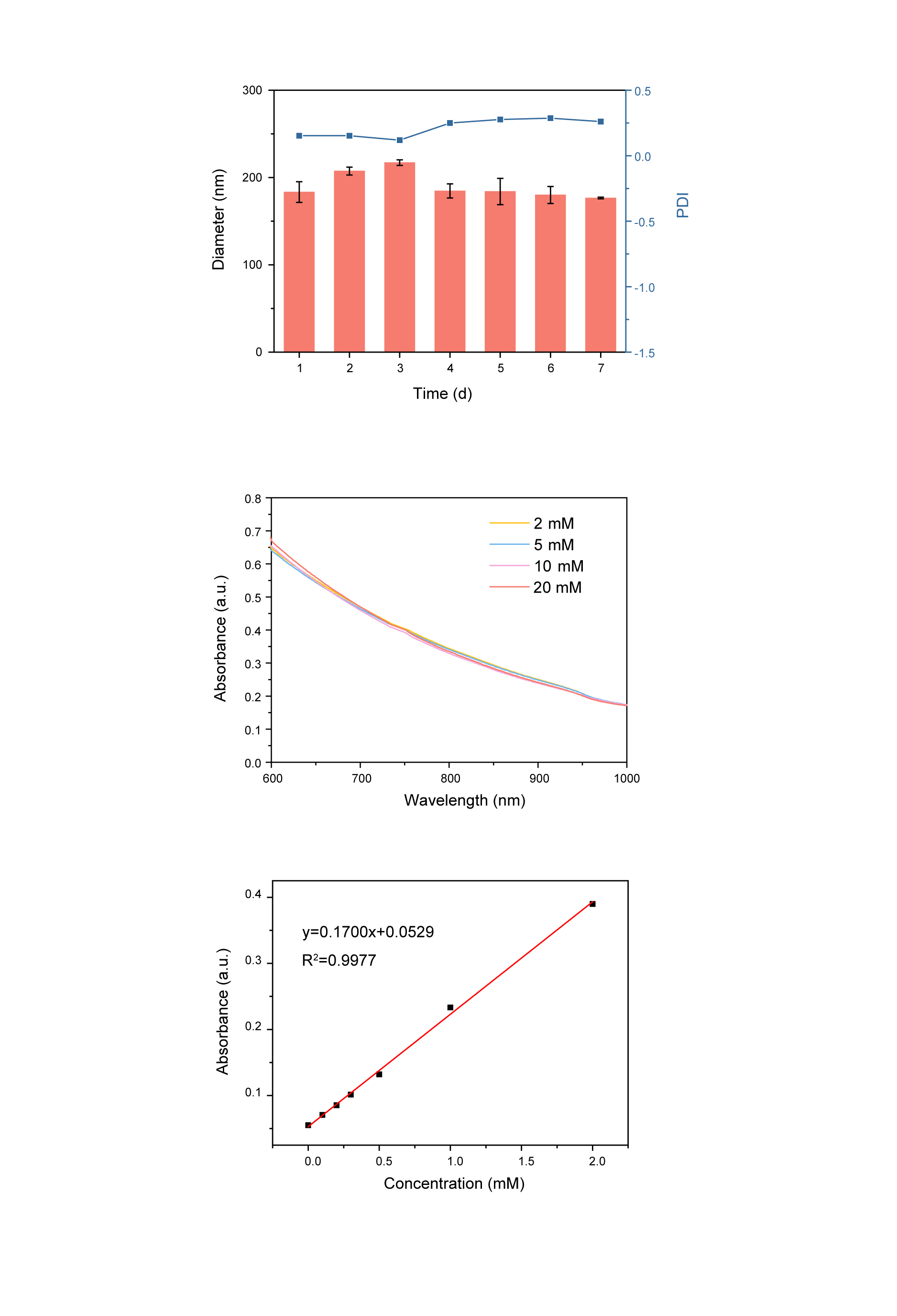


**Figure S16.** UV-Vis spectra of HPP/TPEN@GC after incubated with different concentrations of glucose.


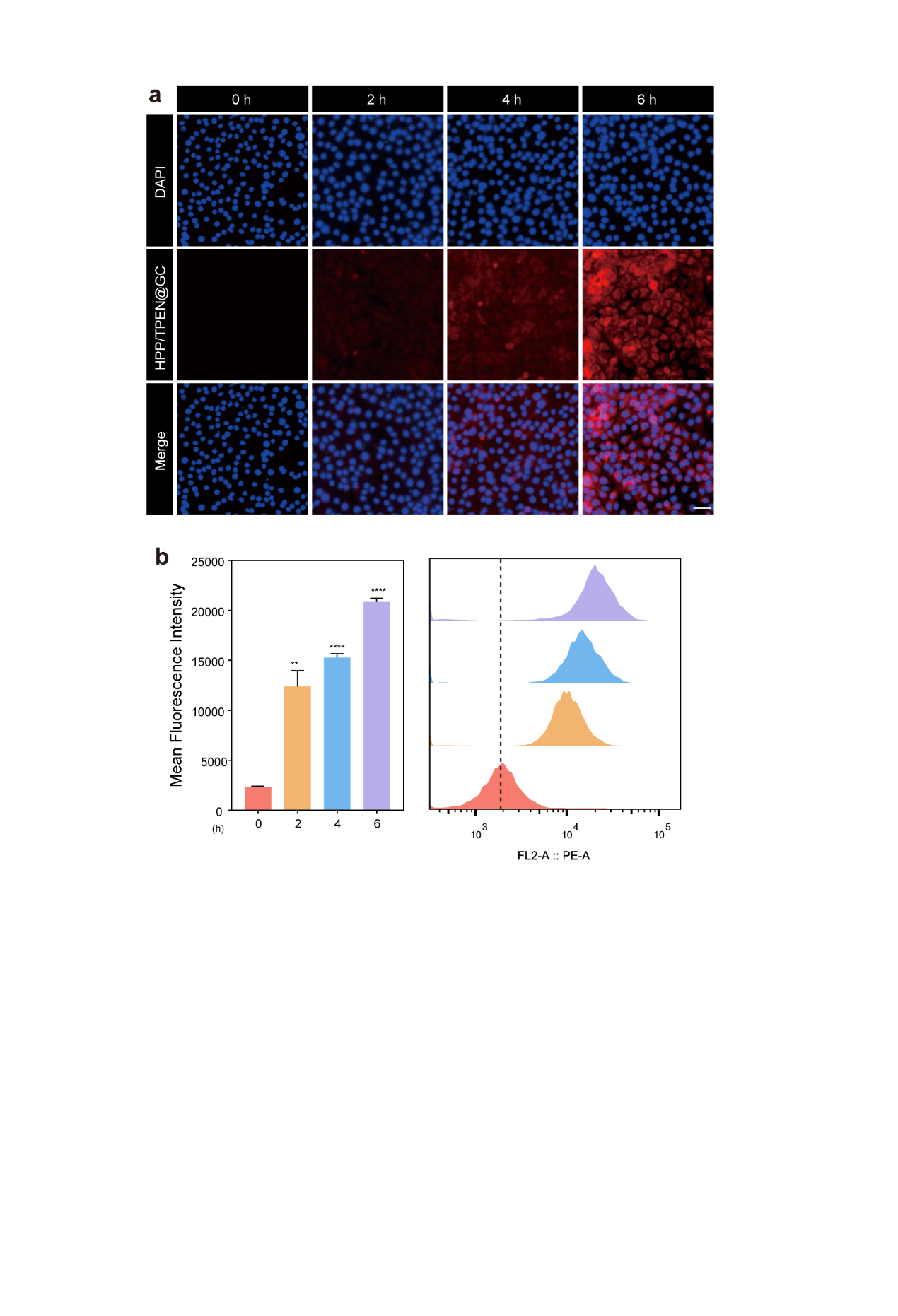


**Figure S17.** (a) Fluorescence microscopy images and (b) flow cytometry analysis of EMT-6 cells cultured with 10 𝜇g/mL RhB-labeled HPP/TPEN@GC for 0-6 h. Scales: 50 𝜇m. **p<0.01, ****p<0.0001 versus PBS group.


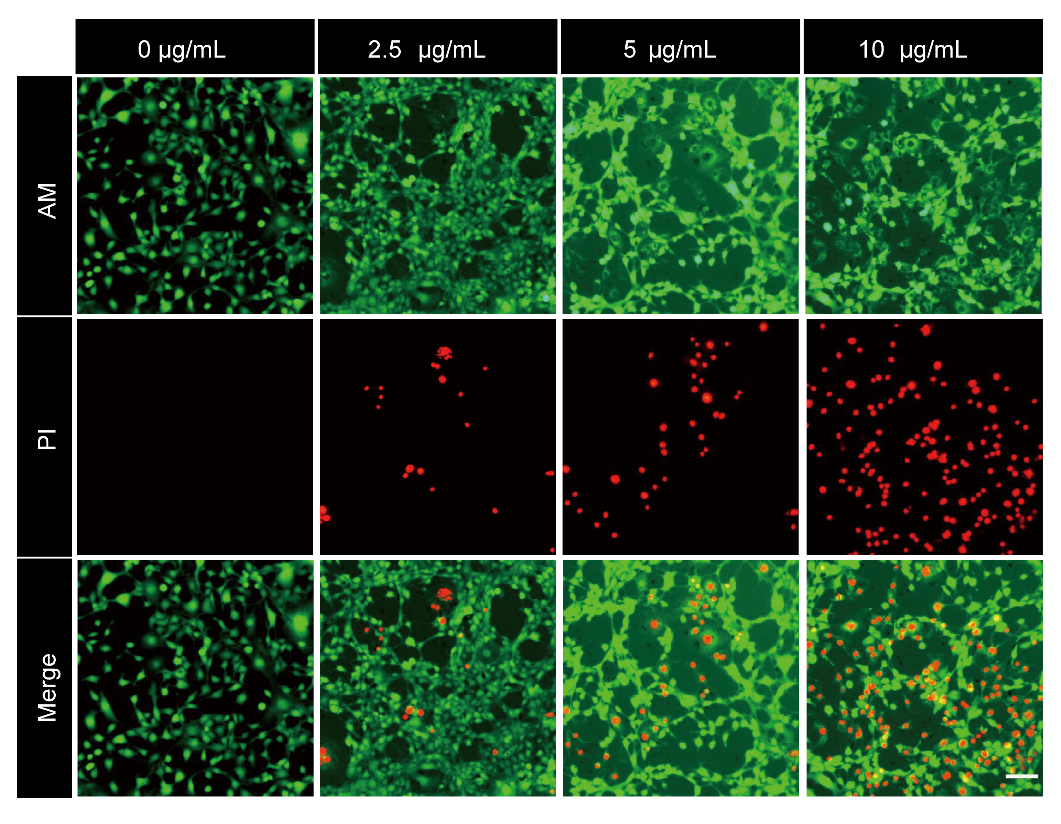


**Figure S18.** The live (green)/dead (red) dual staining images of EMT-6 cells incubated with different concentrations of HPP/TPEN@GC for 24 h. Scales: 50 𝜇m.


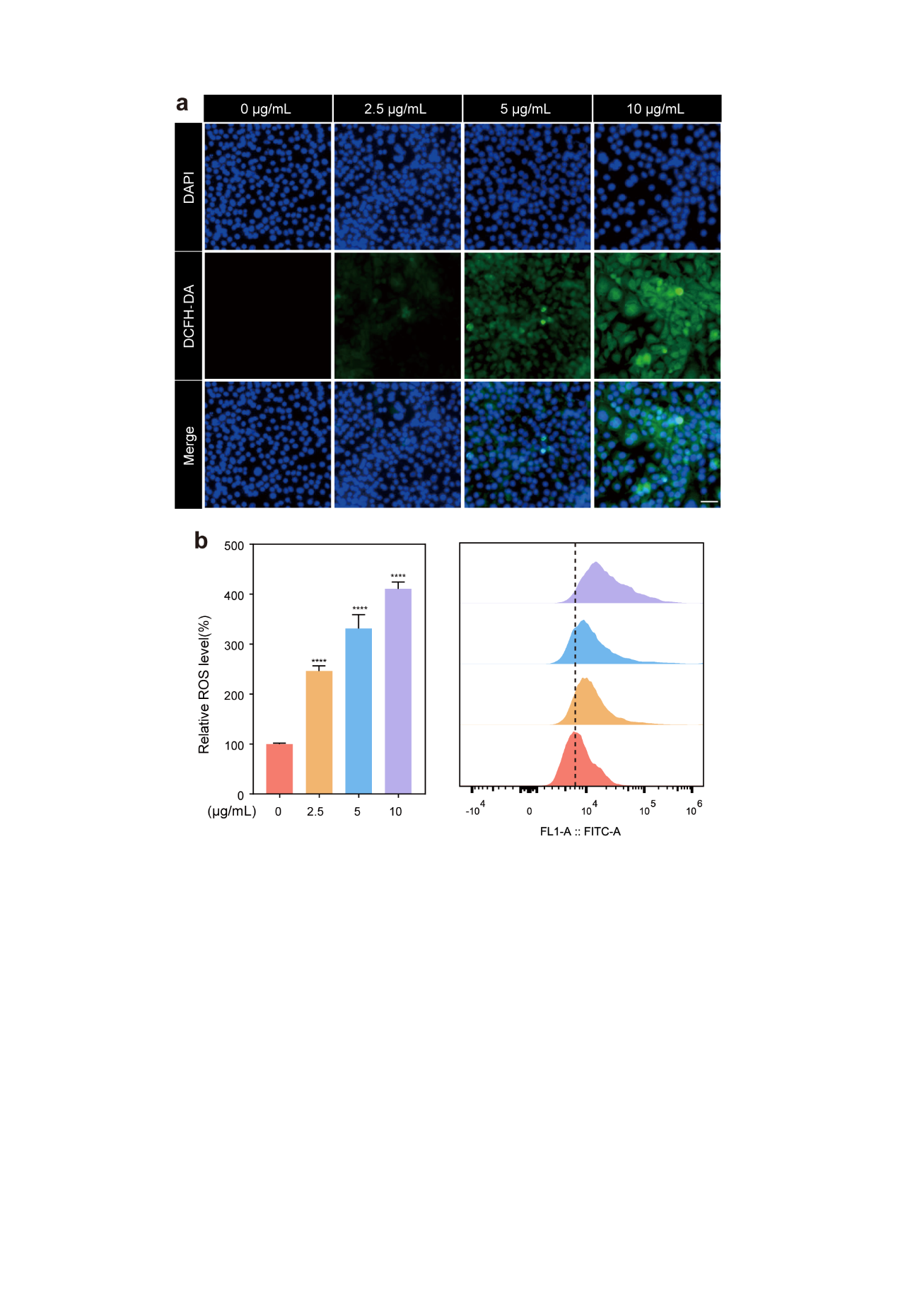


**Figure S19.** (a) Fluorescence microscopy images and (b) flow cytometry analysis of ROS generation after incubation of different concentrations of HPP/TPEN@GC for 4 h. Scales: 50 𝜇m. ***p<0.001, ****p<0.0001 versus PBS group.


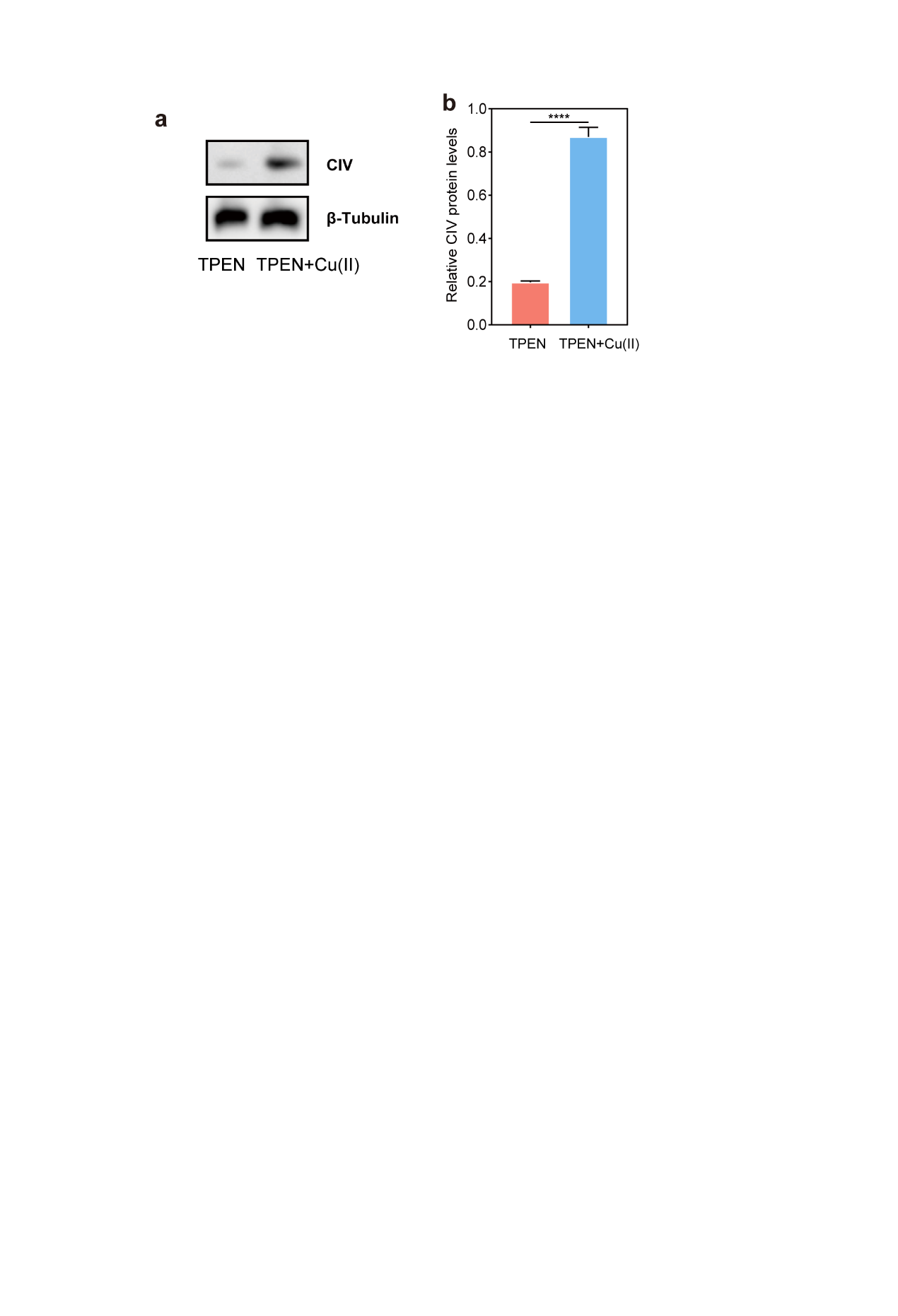
**Figure S20.** (a) Western blot results and (b) corresponding statistical data of Complex IV (CIV) in 5μM TPEN and 5 μM TPEN+10 μM Cu (II) treated EMT-6 cells. Complex IV is referred to as CIV. β-Tubulin was used as a control.


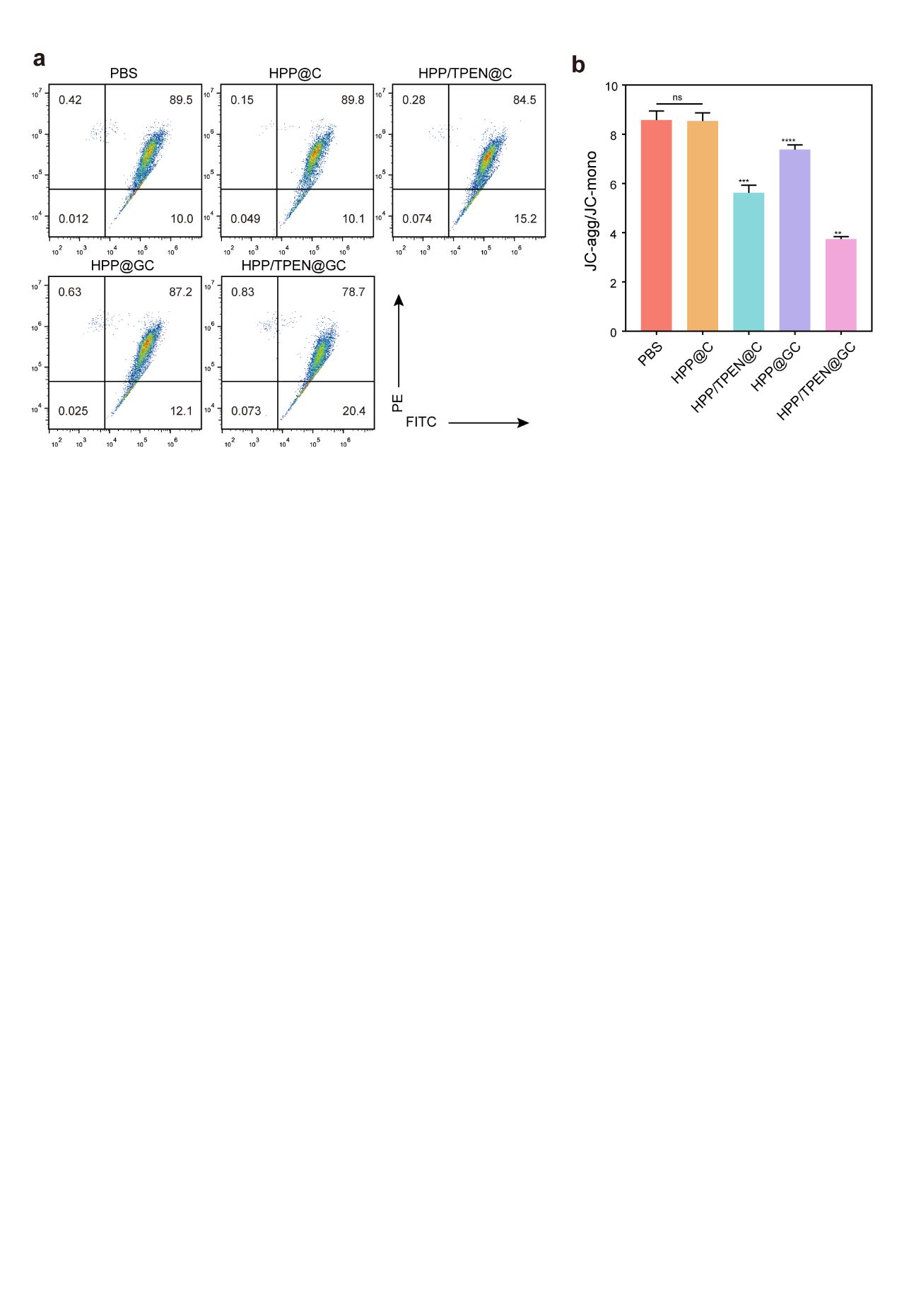


**Figure S21.** (a) Flow cytometric results of mitochondrial membrane potential (MMP) and (b) quantitative analysis of JC-agg/JC-mono of EMT-6 cells treated as indicated using JC-1 dye. MMP is evaluated based on ratio of JC-1 aggregates (JC-agg, red fluorescence)/JC-1 monomers (JC-mono, green fluorescence). **p<0.01, ***p<0.001, ****p<0.0001 versus PBS group; ns, not significant.


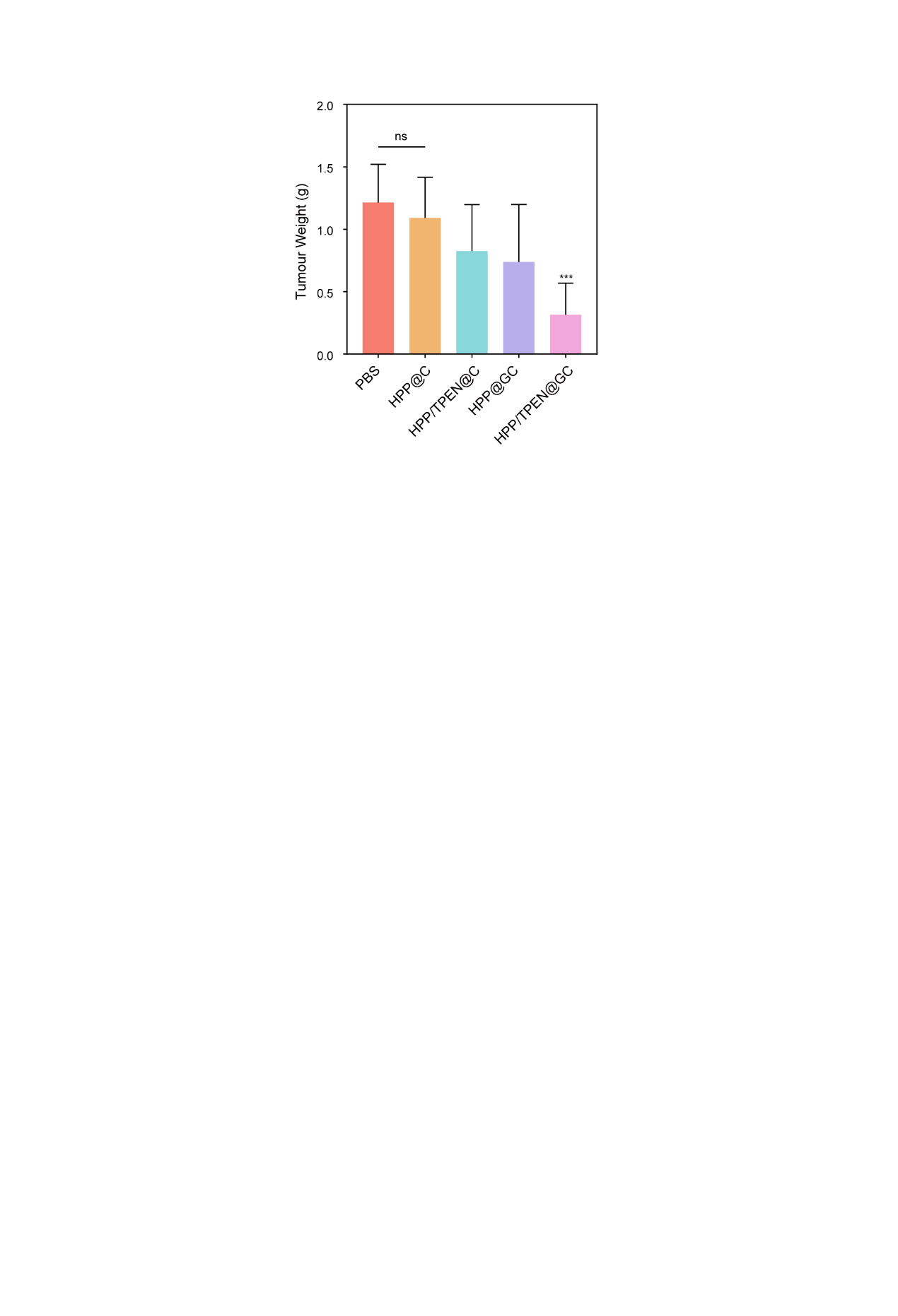


**Figure S22.** The average tumor weight in different groups after treatments. ***p<0.001 versus PBS group; ns, not significant.
